# Quality of care evaluation in non-functioning pituitary adenoma with chiasm compression: visual outcomes and timing of intervention clinical recommendations based on a systematic literature review and cohort study

**DOI:** 10.1007/s11102-020-01044-0

**Published:** 2020-05-18

**Authors:** Iris C. M. Pelsma, Marco J. T. Verstegen, Friso de Vries, Irene C. Notting, Marike L. D. Broekman, Wouter R. van Furth, Nienke R. Biermasz, Alberto M. Pereira

**Affiliations:** 1 Division of Endocrinology, Department of Medicine, Leiden University Medical Center, Albinusdreef 2, 2333 ZA Leiden, The Netherlands; 2 Department of Neurosurgery, Leiden University Medical Center, Leiden, The Netherlands; 3 Department of Ophthalmology, Leiden University Medical Center, Leiden, The Netherlands; 4 Center for Endocrine Tumors Leiden, Leiden University Medical Center, Leiden, The Netherlands; 5 Department of Neurosurgery, Haaglanden Medical Center, Den Haag, The Netherlands

**Keywords:** Non-functioning pituitary adenoma, Pituitary tumor, Transsphenoidal surgery, Optic chiasm compression, Visual outcome, Visual fields

## Abstract

**Purpose:**

Surgery in patients with non-functioning pituitary macroadenomas (NFMA) is effective in ameliorating visual function. The urgency for decompression, and preferred timing of surgery related to the preoperative severity of dysfunction is unknown.

**Methods:**

Systematic review for evidence to provide clinical guidance for timing of surgical decompression of the optic chiasm, and a cohort study of 30 NFMA patients, in whom mean deviation (MD), and severity of visual dysfunction was assessed.

**Results:**

*Systematic review* 44 studies were included with a total of 4789 patients. Postoperatively, visual field defects improved in 87.0% of patients, stabilized in 12.8% and worsened in 1.0%. Specific protocols regarding timing of surgery were not reported. Only seven studies (16.7%) reported on either the duration of visual symptoms, or diagnostic, or treatment delay.

*Cohort study* 30 NFMA patients (50% female, 60 eyes, mean age 58.5 ± 14.8 years), had a median MD of − 5.3 decibel (IQR − 3.1 to − 10.1). MD was strongly correlated with clinical severity (r =  − 0.94, P < 0.0001), and were used for severity of defects cut-off values: (1) normal >  − 2 dB, (2) mild − 2 dB to − 4 dB, (3) moderate − 4 to − 8 dB, (4) severe − 8 to − 17 dB, (5) very severe <  − 17 dB.

**Conclusion:**

Surgical decompression is highly effective in improving visual function. Uniform, quantitative grading of visual dysfunction was lacking. MD is a promising quantitative outcome measure. We provide recommendations for the evaluation of timing of surgery, considering severity of visual impairment, which will need further validation based on expert clinical practice.

**Electronic supplementary material:**

The online version of this article (10.1007/s11102-020-01044-0) contains supplementary material, which is available to authorized users.

## Introduction

Non-functioning macroadenomas (NFMA) are the most prevalent pituitary macroadenomas (25%), and are often diagnosed due to pituitary insufficiency (80%) or visual impairment (85%) [1–3]. Mostly, their growth velocity is slow, but highly variable and therefore unpredictable [4]. NFMAs with suprasellar extension will eventually compress the optic chiasm, -nerves and -tracts, and impair visual function.

Following compression of the optic chiasm, the sequential order of development of visual field defects (VFD) is typical: first in the temporal upper quadrant(s), followed by the temporal lower quadrant(s). Subsequently, defects in the nasal upper and lower quadrants appear, often accompanied by deterioration of visual acuity (VA) and, if no intervention is provided, this can ultimately result in blindness. As the central aspect of the optic chiasm is consistently exposed to higher pressures than the temporal aspects, the intersecting fibers are affected more severely than non-intersecting fibers, leading to the typical bi-temporal VFDs [5]. By contrast, 20% of patients follow a different pattern of visual field (VF) disturbances, because of an alternative configuration of the chiasm (i.e. pre- or postfixed chiasm) [6] or asymmetrical tumor growth. The postoperative recovery of VFDs and, to a lesser extent, of VA is generally considered to be very good [7]. The duration and degree of pressure on the nerve fibers, for example due to growth velocity, are considered to determine the degree of loss of function [8].

Experts agree that in cases of diminished VA, and severe VFDs immediate surgical decompression is required. Presentation with, or progression of, milder VFDs, and mild deterioration of VA are ‘semi-urgent’, and ‘urgent’ indications for surgical intervention, aiming at restoration of visual function whilst preserving (residual) pituitary function [9]. Tumor growth in vicinity of the chiasm and the presence of radiological displacement of the optic chiasm on MR imaging without VFDs are considered ‘non-urgent’ or’elective’ indications for surgical decompression. To date, there is no consensus on the accepted delay for these indications. Many patients will present with a certain diagnostic delay. However, the time between diagnosis and intervention, i.e. treatment delay, is a potentially modifiable factor that could affect postoperative outcome. In the present era of centralization of complex surgical procedures, treatment delay could be an important part of clinical benchmarking of the pituitary center of excellence (PTCOE), and patient referral guidance in its surrounding network. However, the extent of the effects of delaying decompression of the optic chiasm on visual functioning depending on severity of compromised function, and undesirable treatment delay in high-risk patient groups remains unknown to date.

Moreover, VFDs in pituitary disease have typically been described as a qualitative or semi-quantitative measure, somehow subject to individual interpretation. In other ophthalmological diseases, such as glaucoma, the mainstay of reporting severity of disease has become the mean deviation (MD). As provided by automated perimetry, MD is a numerical value in decibel (dB) representing deviation of the VF from age-matched controls, and could therefore be a quantitative measure of severity of VFDs [10]. Note, when VFD progress, the MD becomes more negative. To date, MD has been scarcely used to describe severity of VFDs in studies on NFMA patients [11].

The clinical relevance of prevention of visual morbidity as a benchmark for quality of care in pituitary adenoma patients is highly acknowledged [12]. The aim of this manuscript is to provide clinical recommendations for timing of surgical decompression, based on best available evidence and with a new approach to use MD to improve objective reporting of VFDs severity.

## Methods

### Systematic review

First, we performed a systematic review on the topic of timing of surgical intervention in NFMA patients, adhering to PRISMA guidelines [13].

#### Search strategy

A literature search strategy was developed using medical subject headings (MeSH) and text words related to surgical treatment of NFMA, visual parameters and function tests and timing variables (see Supplementary File). The following databases were searched: PubMed, EMBASE, Web of Science and the Cochrane Central Register of Controlled Trials.

#### Study selection

Randomized controlled trials (RCTs), controlled clinical trials (CCTs), prospective and retrospective comparative cohort studies and case–control studies were included for initial screening. Studies were eligible for inclusion if: (1) the article was published in English; (2) the reported cohort consisted of patients with NFMA; (3) the reported study cohort consisted of at least 10 patients; (4) patients were treated surgically with transsphenoidal microscopic, or endoscopic techniques, or transcranial techniques; (5) visual parameters were assessed prior to and after surgery. Studies on patients with various types of pituitary adenomas were excluded if the published results were not stratified by pituitary tumor type. Reviews, letters to the editor and expert opinions were excluded. Two independent reviewers (ICMP and MJTV) assessed eligibility and disagreements were resolved by discussion and consensus.

#### Quality assessment

An assessment was formulated by our research group to assess the quality of the included studies. Fourteen items were defined, including timing of surgery and visual function test, pre- and post-operative magnetic resonance imaging (MRI) and visual function characteristics and follow-up time (Supplementary Table 1). Individual scores ranged from 0 to 34 points. The median of the quality score percentages was used as a cut-off point for low- and high-quality papers. Studies were not in- or excluded based on the quality assessment.

#### Data extraction

The following data were extracted: sample size, gender distribution, age, adenoma characteristics, timing of surgery, surgical technique, surgical outcome, visual function and the timing of visual diagnostics. Factors described in two studies or more were considered eligible for inclusion in the systematic review. Unfortunately, the reporting of the outcome of similar variables varied greatly amongst studies. Therefore, all parameters described in this review were (re)defined by the authors. Following data extraction, lead authors were contacted regarding remaining questions, when applicable.

### Cohort study: evaluation of severity of VFDs using MD

#### Patient selection

To validate the use of MD instead of a subjective semi-quantitative description of severity of VFD (e.g. mild, moderate, or severe, quadrantanopia, or hemianopia), a cohort of patients with an NFMA operated in our center followed in our Value Based Health Care (VHBC) care path with a dedicated clinical and patient-reported outcome set (2016–2018) was selected [14]. Patients with ocular or neurological comorbidities, such as glaucoma, retinitis, or MS, were excluded from this study population.

#### Collection of data

For all patients, age, adenoma characteristics and the outcome of pre- and postoperative visual function tests were recorded. Treatment delay was calculated using the date of diagnosis of VFD and the date of surgery. All included patients were studied using perimetry performed with an automated central 30–2 Humphrey field analyzer (HFA) (Carl Zeiss Meditec, Jena, Germany) in the presence of an ophthalmic technician. From the perimetry assessment, MD and Visual Flied Index (VFI) were recorded for each eye individually.

#### Blind comparison of physicians’ interpretation and MD

From our multidisciplinary team (MDT), eight treating physicians were selected: two neuro-ophthalmologists, one neuro-ophthalmologist in training, one neuro-endocrinologist, one endocrinologist-in-training, and three pituitary neurosurgeons. All physicians are specialized in pituitary disease and set the indication and time frame for surgical decompression in the clinical care setting.

Using a standardized survey, we assessed the physician’s view on the pattern and severity of VFD based on HFA assessment blinded for MD and VFI. Since all physicians were very proficient in Dutch, the questionnaire was in Dutch. The questionnaire started with one general question regarding their assessment of the HFA assessment (such as fixation losses, false negatives, and false positives). Next, the individual eyes were scored for pattern and severity of VFD. Rating of patterns was scored as follows (1) no defects, (2) defects due to glasses etc., (3) quadrantanopia (4) temporal hemianopia, (5) superior hemianopia (6) inferior hemianopia, (7) nasal hemianopia and, (8) other. Rating of the severity was scored as follows: low (0 points), mild (1 point), moderate (2 points), severe (3 points), very severe (4 points). Based on the assessment of the two eyes of one individual, recommendation of the indication and timing of surgical decompression (i.e. not indicated, within 3 days, 1 week, 2 weeks, 4 weeks, 6 weeks or 3 months).

For the severity, average severity scores of all eight assessing physicians (of whom three were ophthalmologists) were calculated. Additionally, average severity scores were calculated for the ophthalmologists only. Following the averaging of the scores, patients were divided into severity categories. Scores from 0 to 0.5 were considered low, 0.5 to 1.5 mild, 1.5 to 2.5 moderate, 2.5–3.5 severe and 3.5 and higher very severe. These obtained scores were compared to and associated with MD.

### Statistical analysis

All data was collected using Microsoft Excel (Microsoft Corporation, Redmond, WA, USA) and graphs were made using GraphPad Prism 7 (GraphPad Software, La Jolla, CA, USA). For the systematic review, individual study data are depicted as median (interquartile range (IQR)) or n (%) unless otherwise specified. No comparative statistical analyses were performed because of the heterogeneity of the outcome data. For the cohort study, all data were tested for normal distribution and reported as mean ± SD or median (IQR), when applicable. Intra-class correlations (ICC) were calculated using Reliability Analysis. Spearman’s correlations were performed for severity scores and MD.

## Results

### Systematic review

#### Study and patient characteristics

The search conducted in December 2019 yielded 3322 articles, of which 3068 articles were excluded based on title and abstract (See Supplementary Fig. 1 for the inclusion, and exclusion process). Ultimately, 44 articles were included [7, 15–57]. As shown in Table 1, out of the 44 included articles, 30 reported on NFMA patients only. Only four studies (9.1%) were prospective cohort studies [18, 22, 33, 56], whereas the remaining 40 studies (90.9%) were retrospective cohort studies. A total of 4789 patients were included, and patients characteristics appear representative of the general NFMA patient population (Table 1). Of note, thirty articles (68.2%) reported on transsphenoidal surgery (TSS), 2 articles (4.5%) reported on transcranial surgery (TCS), 7 articles (15.9%) reported on both TSS and TCS, and 5 articles (11.4%) did not report on the type of surgery.

**Table 1:** General characteristics of the included articles and study population

General study and patient characteristics			
Studies	Number included studies	All	44
		Reporting on NFMA only	30 (68.2%)
	Study design	Prospective cohort	4 (9.1%)
		Retrospective cohort	40 (92.9%)
	Publication dates		1983–2019
	Study period		1971–2018
Patients	Number included patients		4789
	Patients per study		72.5 (35.0–123.3)
	Age (years)^a^		55.0 (49.6–58.5)
	Male patients^b^		2778 (60.9%)
	Surgical approach	Transsphenoidal	30 (68.2%)
		Transcranial	2 (4.5%)
		Combined	7 (15.9%)
		Unknown	5 (11.4%)
	Follow-up duration (months)^c^		43.8 (14.0–61.4)

Data are shown as N, N (%), or median (IQR) unless otherwise specified

*N* number of articles or patients

^a^Age was reported in 41/44 articles

^b^Gender was reported in 43/44 articles

^c^Follow-up duration was reported in 38/44 articles

#### Quality assessment

All 44 articles were assessed using the Quality Assessment Tool (QAT), of which the results are shown in Supplementary Table 2. Twenty-two studies (50.0%) were classified as high-quality studies (> 39.7%). Of the QAT, three items were most important for the quality of this review: reported timing of surgery, pre-operative visual function tests, and postoperative visual function tests. Timing of surgery scores were low, as solely three studies reported this factor (sub-scores ranging from 1 to 3).

#### Prior and post treatment evaluation of VF and VA

As depicted in Supplementary Fig. 2a, preoperative VFDs were reported in 69.0% (median, IQR 62.5–93.0%) of 2305 patients (48.1% of included population) in 27 studies (61.4%) [7, 15, 16, 18, 19, 21, 23–31, 34–36, 38, 40, 41, 43, 45, 48–50, 53, 55]. Severity of preoperative VFDs was reported in 9 studies (20.5%) only, which are shown in Table 2 [18, 19, 24–26, 34, 35, 48, 55]. Pre- and postoperative VFDs were reported for 1,300 NFMA patients (27.1% of included population), of which 72.0% (median, IQR 65.8–92.5%) of patients were reported to have preoperative VFD, in 16 articles (36.4% of included studies) (Supplementary Fig. 2b) [15, 16, 18, 19, 21, 24, 26, 27, 29, 31, 40, 41, 43, 49, 50, 55]. Postoperatively, VFDs were recorded as improved in 87.0% of patients (median, IQR 73.8–92.3%), stabilized in 12.8% (median, IQR 1.5–17.0%) and, worsened in 1.0% (median, IQR 0.0–4.0%) (Supplementary Fig. 2c). Unfortunately, none of the included articles mentioned MD of the patient cohort. Furthermore, outcome data could not be grouped based on pre-operative severity.

**Table 2:** Severity of pre-operative visual field defects, visual acuity impairment and parameters of timing

Assessment	Author	Participants	Pre-operative defects	Severity of pre-operative defects
Visual fields	Berkmann et al. [18]	26	96	Normal VF N = 1; Quadrantanopia N = 12; Hemianopia N = 13
	Berkmann et al. [19]	85	69	Normal VF N = 26; Quadrantanopia N = 18; Hemianopia N = 41
	Colao et al. [24]	84	36.9	Normal VF N = 53, Quadrantanopia N = 7; Hemianopia N = 26
	Dallapiazza et al. [25]	80	52	Normal VF N = 38 Unilateral hemianopia N = 12; Bitemporal hemianopia N = 22; Other N = 9
	Dekkers et al. [9]*	43	90.7	Normal VF N = 4; Mild defects N = 7; Moderate defects N = 9; Severe defects N = 23
	Holder et al. [34]	34	100	Symmetrical bitemporal hemianopia N = 16; Asymmetrical bitemporal hemianopia N = 6; Asymmetrical bitemporal hemianopia with paracentral scotoma N = 4; Bitemporal superior quadrant loss N = 2 Bitemporal superior quadrant loss with paracentral scotoma N = 2; Unilateral superior quadrant loss N = 2; Congruous homonymous hemianopia N = 2; Severe generalized loss in one eye with temporal field loss in the other N = 4
	Jahangiri et al. [35]	75	100	Bitemporal hemianopia N = 30; Difficult to define N = 19; Uniocular N = 12; Quadrantanopia in one eye combined with a quadrantanopia or hemianopia in the other eye N = 8; Missing N = 6
	Sheehan et al. [48]**	70	84.3	Visual Field Index; Endoscopic group 3.5 (1–4); Microscopic group 3.0 (1–4)
	Zhang et al. [55]	208	96	Normal VF N = 8; Bitemporal hemianopia N = 157; Superior quadrantanopia N = 24; Unilateral temporal hemianopia with contralateral blindness N = 19
Visual acuity	Berkmann et al. [18]*	26	92	Normal VA N = 2; Slightly decreased VA N = 9; Moderately decreased VA N = 11; Severely decreased VA N = 4
	Colao et al. [24]	84	32	Normal N = 57; Unilateral partial loss N = 9; Bilateral partial loss N = 11; Dimming of eyesight N = 7
	Holder et al. [34]	34	77.9	Normal VA 15 eyes; 6/9 13 eyes; 6/12 9 eyes; 6/18 12 eyes; 6/24 7 eyes; 6/36 3 eyes; 6/60 3 eyes; < 6/60 6 eyes
	Trautmann et al. [50]	226	41.4	Not tested N = 11; 20/20–20/40 N = 126; 20/50–20/200 N = 52; < 20/200 N = 37
	Zhang et al. [55]	208	99	Normal VA N = 3; Unilateral impairment N = 54; Bilateral impairment in N = 151
Timing of surgery				
Timing of surgery	Anagnostis et al. [16]	114		Mean 3.7 ± 1 months (range 1–48 months)
	Berkmann et al. [18]	32		Mean 14.9 ± 19.5 weeks
	Jahangiri et al. [35]	75		Range 1–29 days
Diagnostic delay	Marenco et al. [41]	25		Range 6 months–8 years
	van Lindert et al. [51]	53		Mean 3.3 ± 5.0 years (range 0–18 years)
Symptom duration	Holder et al. [34]	34		Mean 16 months (range 1 week–4 years)
	Jahangiri et al. [35]	75		Median 6.5 months (range 1 week–15 years)
	Nakao et al. [45]	43		Mean 14.9 months (range 2–40 months)

Data are shown as percentage affected patients reported per study

*N* number of patients, *VA* visual acuity, *VAI* impairment of visual acuity, *VF* visual field, *VFD* visual field defects

*Visual fields* *VFD classification as described by Dekkers et al. [26]: mild, if there were peripheral defects in only one quadrant; moderate, if the upper quadrants were affected; severe, if combined upper and lower quadrant field defects. **Visual Field Index, as described by Sheehan et al. [48]: 0, completely normal perimetry results or imaging studies showing no encroachment of the optic chiasma; 1, any defect less than a quadrantanopia in either eye or both eyes (mild); 2, a quadrantanopia in either eye or both eyes (moderate); 3, any defect greater than a quadrantanopia in either eye or both eyes (severe). *Visual acuity* *VA classification as described by Berkmann et al. [18]: slight, 15–40% loss of central vision; moderate, 40–70% loss of central vision; severe, > 80% loss of central vision; and absent, 100% loss of central vision. In cases involving asymmetrical visual acuity, the more impaired eye was referred to. *Timing of surgery* Timing of surgery is defined as the time from diagnosis to surgery, i.e. treatment delay. Diagnostic delay is defined as the time between onset of symptoms and diagnosis

Preoperative impairment of VA was reported in 51.0% (median, IQR 32.5–76.2%) of 1354 patients (28.3% of included population) in 12 studies (27.3%), as depicted in Supplementary Fig. 2d [18, 19, 23, 24, 28, 34, 38, 40, 43, 50, 53, 55]. Severity of preoperative impairment of VA was reported in 5 studies with 5 different grading systems (Table 2) [18, 24, 34, 50, 55]. Pre- and postoperative VA was reported for 662 NFMA patients (13.8% of included population), of which 46.6% (median, IQR 30.6–66.0%) of patients were reported to have preoperative impairments, in 6 articles (13.6% of included articles) (Supplementary Fig. 2e) [19, 24, 40, 43, 50, 55]. Postoperatively, VA was recorded as improved in 91.0% of patients (median, IQR 68.8–98.5%), stabilized in 5.5% (median, IQR 1.5–27.6%) and, worsened in 1.0% (median, IQR 0.0–2.8%) (Supplementary Fig. 2f). Again, baseline characteristics could not be linked to outcome data.

#### Additional visual assessment parameters

With the exception of Visual Impairment Score (VIS) and Visual Evoked Potentials (VEPs), no other visual function parameters were assessed in the included articles. Nakao et al. reported a preoperative VIS of 73.1 ± 18.4, which improved to 41.7 ± 18.8 postoperatively [45]. Watanabe et al. reported a pre- and postoperative VIS in younger (< 70 years) and older patients (≥ 70 years), which were lower overall in older patients, and showed similar improvement rates in VIS following surgical resection of the adenoma (56.2% younger patients with improved scores vs. 46.9% of older patients) [57]. Holder et al. assessed VEP preoperatively in 34 patients, in which 100% of VEPs were abnormal [34].

#### Timing of surgical intervention or diagnosis

None of the 44 included articles provided details of protocols or policies regarding timing of surgery in relation to severity of visual symptoms. As depicted in Table 2, only 7 studies (15.9%) reported on either the duration of visual symptoms, the timing of diagnosis, or surgery. One study prospectively collected data on timing [34], the other data were collected retrospectively. Of note, Berkmann et al. described a mean duration of 14.9 ± 19.5 weeks from start of symptoms to surgical intervention in 32 patients, of which 26 with non-functioning pituitary tumors [18], and Marenco et al. reported that in a cohort of 25 elderly patients (> 65 years) the symptom duration varied from 6 months to 8 years until diagnosis [41].

When assessing the effects of time-dependent parameters, and surgical outcome, three studies stated that earlier surgical intervention improved postoperative results. Jahangiri et al. reported that patients with complete recovery (CR) had a median symptom duration of 3.5 months, whereas the median symptom duration was 12 months in patients with partial recovery (PR) [35]. Nakao et al*.* stated that duration of visual symptoms significantly affected visual outcome based on the recovery rates in the following patient groups: CR in 90% patients and PR in 10% of patients with symptom duration < 6 months, CR in 54.5% of patients and PR in 45.5% of patients with symptom duration 6–12 months and, CR in 5% of patients and PR in 95% of patients with symptom duration > 12 months [45]. Furthermore, Chen et al. reported prospectively collected timing of surgery for 37 clinically apoplectic patients in the cohort (9.6% of total study population), of which 32 patients had visual dysfunction [22]. Treatment delays ranged from 6 h to 7 days, and percentages of resolution of impaired visual function were higher in patients operated within 3 days as compared to those operated after 4 to 7 days.

#### Other influencing factors on postoperative outcome of visual function

Decompression of the chiasm on intraoperative imaging, surgical experience, age, and tumor size were reported as significant risk or protective factors in 7 of 44 articles (15.9%). Berkmann et al. reported that a decompressed optic chiasm on intra-operative MR imaging was correlated with postoperative VF and VA improvement [18]. Magro et al*.* and Mortini et al*.* described improved outcomes with increased surgical experience [40, 44]. Moreover, younger patients had a higher chance of post-surgical improvement of visual function [33, 43]. By contrast, Jahangiri et al*.* reported that age at diagnosis was not predictive for recovery rates, although age correlated with increased symptom duration [35]. Additionally, the severity of preoperative optic nerve fiber degeneration, i.e. optic disc pallor, was linked to the lack of visual normalization [20].

#### Exemplary cases of adverse visual outcome from literature

Of the 44 included studies, 7 specifically reported on patients with deterioration of visual function. Ferreli et al. stated that the surgical procedure was efficacious and safe with only one patient experiencing deterioration of visual acuity postoperatively [28]. In a series of 300 NFMA patients, postoperative transient and permanent visual worsening were reported in 3% and 2.4% of cases, respectively [40]. Most worsening was related to postoperative intrasellar hematoma and remained unexplained in the 5 remaining cases. In addition, Dallapiazza et al. and Mortini et al. reported that postoperative hematoma causing loss in visual function could occur [25, 44]. Permanent worsening of visual acuity in 1 eye was reported, possibly caused by operative manipulation and a large suprasellar remnant, which did not improve after a second operation 4 months later, regardless of total removal of the remnant [37]. Watanabe et al. reported deterioration in one patient with hemorrhaging and in one patient following surgical manipulation [57]. Holder et al. reported that after a follow-up duration of several years, some patients experienced a rapid deterioration in vision without an apoplectic event [34].

### VFD patterns, and quantitative evaluation by MD in pilot cohort of 30 NFMA patients

Of the 30 studied patients, 15 were female (50%) and the mean age was 58.5 ± 14.8 years. Two patients had a giant adenoma (maximum diameter > 4 cm), and the remaining 28 patients had a macroadenoma (maximum diameter 1-4 cm). All 60 eyes were assessed, of which individual outcomes of the visual function tests are shown in Supplementary Table 3. Median MD was − 5.3 decibel (dB) (IQR − 3.1 to − 10.1) and median VFI was 91.5 (IQR 67.3–96.0).

Reliability for pattern determination as assessed by ophthalmological physicians was low, with an ICC of 0.37 (95% CI 0.27–0.49). By contrast, for the severity scores as assessed by all physicians, the reliability was high, with an ICC of 0.81 (95% CI 0.74–0.87). Therefore, severity scores were averaged for all physicians, which were highly correlated with the MD of the 60 eyes (r =  − 0.94, P < 0.0001). Following the averaging of the severity scores, patients were divided into the aforementioned severity categories. Severity grading was recorded as low in 7 eyes, mild in 20 eyes, moderate in 13 eyes, severe in 14 eyes, and very severe in 6 eyes. MD for the separate severity categories were assessed, and scores until − 2 dB were considered normal. MD for mild defects ranged from − 2 to − 4 dB, MD for moderate defects ranged from − 4 to − 8 dB, and MD for severe defects ranged from − 8 to − 17 dB. MD below − 17 dB were considered very severe.

**Fig. 1:**
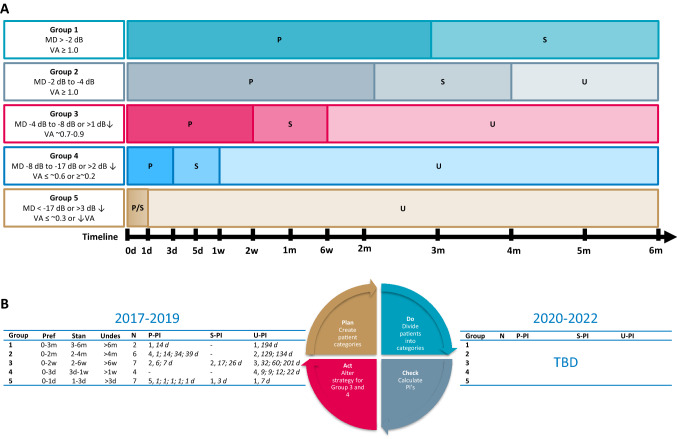
Recommendation for timing of surgery depending on visual function and compression of optic chiasm and evaluation of NFMA patient cohort. **a** Timeframes for surgical intervention are divided into three categories: preferred (P), standard (S) and undesirable (U). In case of progression of clinical symptoms, upgrade to other patient group and advance surgical intervention. During surgical delay, VF and VA testing should be repeated according to ophthalmological FU timeframes: Group 1, every 3 months; Group 2, every 4 to 6 weeks; Group 3, every 1 to 3 weeks; Group 4, every 3 to 5 days. **b** Data are shown as number of patients (N) operated within the preferred, standard and undesirable timeframes as suggested in section **a**. Treatment delay (in days) is reported per patients in *Italics*. Performance indicators (PIs) were calculated for preferred (P-PI), standard (S-PI), and undesirable (U-PI) timeframes. *FU* follow-up, *VF* visual fields, *VFD* visual field defects, *VA* visual acuity, *d* day(s), *w* week(s), *m* month(s), *D* days, *NFMA* non-functioning adenoma, *VFD* visual field defects

Of the 30 patients, four patients with radiological compression of the chiasm with still normal visual function were operated for other reasons. In the remaining 26 patients, the operation indication was worsening of VFD or VA. Therefore, treatment delay was calculated for these 26 patients, resulting in a median delay of 13 days (IQR 3–35). Patients were divided into the patient categories proposed below, as is shown in Fig. 1b.

## Discussion

Decompression of the optic chiasm is without doubt very effective in improving visual function in the clear majority of patients. Improvement in VFDs and VA is to be expected in 87.0% [15, 16, 18, 19, 21, 24, 26, 27, 29, 31, 40, 41, 43, 49, 50, 55], and 91.0% [19, 24, 40, 43, 50, 55] of patients, respectively. Some studies reported better postoperative outcomes when the delay to decompression is shorter [34, 35, 45], however, time of symptom onset remains a subjective measure, which hampers making conclusions. Visual function deteriorated in a very small subgroup of patients following surgical intervention, for which the main risk factor appeared to be perioperative hemorrhage. Moreover, reversibility of VFD and VA remain very likely despite a significant diagnostic delay in many patients.

A clear limitation of the available literature includes the heterogeneity of the studied population. Patients present with VFDs and impaired VA prior to surgery, in 20–100% [7, 15, 16, 18, 19, 21, 23–31, 34–36, 38, 40, 41, 43, 45, 48–50, 53, 55], and 20–99% [18, 19, 23, 24, 28, 34, 38, 40, 43, 50, 53, 55] of patients, respectively. Furthermore, consensus on grading or stratification of severity of visual dysfunction is lacking. Without uniform description of population and outcome, a meta-analysis of available data couldn’t be performed. Moreover, measurements of visual function in NFMA patients were currently limited to VF and VA [15, 16, 18, 19, 21, 23, 24, 26–29, 31, 34, 38, 40, 41, 43, 49, 50, 53, 55], with solely two studies reporting on VIS and VEP [34, 45]. Some studies reported on non-validated composite scores for visual impairments. Therefore, potential promising visual parameters for prediction of visual outcome, e.g. color vision, optic disc pallor, optical coherence tomography (OCT) and radiological characteristics of the optic chiasm on MRI, were not assessed in the studied population. Description of patient characteristics and outcome measures needs to be improved in future studies, as highlighted in a recent Pituitary Society Position Statement, e.g. clinically relevant visual dysfunction at presentation, and the extent of visual improvement or deterioration in time [58]. Moreover, this is essential for both clinician-reported, and patient-reported outcomes, since improvement of mild VFDs can be clinically insignificant, whereas improvement in VA and severe VFD will overcome thresholds previously impairing daily life [26].

### Evaluation of severity of VFD using MD

Since clear grading systems to quantify the severity of VFD were unavailable, we aimed to provide an easy and objective alternative for reporting on the severity of VFD. For this purpose, we assessed which MD reflects expert-physician-judged severity of VFDs. MD is well known in glaucoma care and research, and recent reports have started to use this promising objective measure in sellar, and suprasellar tumors [11, 59]. Interestingly, different tumors might impact optic chiasm function and visual function tests in a different manner, which hampers the use of simple categorization in’classical’ hemi- and quadrantanopia [60]. With this study in patients with NFMA, we show a clear relation between the current ‘best practice’ of severity reporting, namely the opinion of ophthalmologists regarding the pattern of VFDs, and an quantitative outcome measure of severity, namely MD, including a proposal for clinically applicable cut-off values. Minor pitfalls of using the MD are the learning curve, and concentration of the patient (as reflected by fixation losses, false negatives, and false positives), and the influence of multiple ophthalmological diseases [61]. Therefore, it is important to note that these scores still require expert neuro-ophthalmologist interpretation, may not reflect actual improvement during follow-up, and should be interpreted considering the patient’s age and performance [10, 56]. Nevertheless, MD is a promising tool in the clinical decision-making and outcome evaluation in NFMA patients (vide infra).

### Visual outcomes in relation to timing of surgery

The evidence obtained from the systematic review that a longer duration of symptoms is adversely related to postoperative visual outcome is weak. Duration of symptoms consists of two components: first, time between the start of symptoms and diagnosis (i.e. the diagnostic delay), and second, timing of surgery, defined as the time between diagnosis and surgical intervention (i.e. the treatment delay). Unfortunately, patients might not notice mild loss of peripheral vision, and therefore estimated diagnostic delay is unreliable. Longer symptom duration was related to decreased recovery rates [35, 45], and similar tendencies for multiple pituitary adenoma types have been reported [8, 62]. Several factors were related to worse outcome, namely radiological compression of the chiasm, and adenoma growth velocity [63], and severity of preoperative optic nerve fiber degeneration on OCT [20]. Finally, postoperative recovery of VA was correlated with disease duration in a pituitary macroadenoma cohort [64]. Therefore, it is plausible that timing of surgical intervention is an important determining factor for postoperative visual recovery.

Treatment delay, in contrast to diagnostic delay, is a modifiable factor, but has been scarcely studied. Treatment delay was reported in only three studies, with extremely variable individual timeframes ranging from 1 day to 48 months following NFMA diagnosis, refraining from mentioning outcome and severity of visual dysfunction at presentation [16, 18, 35]. Moreover, postponing surgery for 4 weeks after diagnosing the pituitary adenoma has been reported not to affect postoperative visual acuity outcomes [26]. Unfortunately, the influence of this surgical delay on the development of impairment of VFs in time was not reported. Finally, studies refrained from reporting on protocols regarding preferred timing of surgery in clinical practice. Personal correspondence with the authors of the included articles revealed quite a heterogeneous picture of clinical practice regarding timing of surgery in various patient groups, as well as absence of institutional and international policies. Local practice varied between swift intervention, and waiting list management. Based on the identified limitations of literature (e.g. heterogeneous descriptions of delay, lack of studies on impact of the duration between diagnosis and surgery on outcome, and lack of surgical timing protocols in literature), clinical recommendations regarding timing of surgery cannot be evidence-based at this time. Therefore, prospective data collection with a uniform outcome set is an unmet need. We propose a classification based on severity and progression to provide a first tool for clinical benchmarking, and starting this discussion. Pituitary tumor centers of excellences (PTCOE) should have a leading role in further developing protocols for referral and timing of surgery.

### Considerations for continuous improvement of care for patients with compromised visual function

Ultimately, all patients with an NFMA and concomitant compression of the optic chiasm are candidates for surgical removal of the adenoma. However, consequences of surgical delay will depend on multiple individual factors. In scarcity situations, identification and prioritization of patients at risk for adverse outcomes when treatment is delayed is pivotal. Consequently, uniforms definitions of visual (dys)function and treatment delay are needed. The following clinical recommendations address this clinical unmet need in a stepwise approach, according to the Plan-Do-Check-Act cycle: Plan: (1) Establishment of a patient classification based on severity or progression of visual dysfunction using a uniform clinician- and patient-reported outcome set. (2) Proposal of preferred and undesirable time frames for timing of surgery for each group for clinical benchmarking. Do: (3) Prospective data collection with proposed uniform outcome set and timeframes. Check: (4) Evaluation of data. Act: (5) Refinement of patient classification, outcome set, time frames based on evaluation of prospectively collected data, consensus discussion, and further development by a network of PTCOEs, and implementation in official guidelines.

### Towards clinical benchmarking for NFMA patients with optic chiasm compression

In the current management of pituitary adenomas, an optimal ophthalmological examination is paramount to ensure optimal timing of treatment [12]. Two main examinations for proper patient stratification are the evaluation of VFs and VA. As exemplified by Fig. 1a, we propose 5 categories of pre-operative severity of visual dysfunction, based on MD and VA in agreement with ophthalmological practice: conservative treatment: (0) patients with normal VF and VA testing electing for wait-and-scan approach; surgical treatment: (1) patients with normal VF and VA testing electing for surgery; (2) patients with mild VFD with normal VA; (3) patients with moderate VFD or subtle impairments in VA; (4) patients with severe VFD, impaired VA or progressive deterioration of VA and (5) patients with very severe VFD, extreme loss of VA, or acute severe deterioration of VA. These categories represent distinct patient groups with increased urgency of referral and surgical intervention, and ophthalmological surveillance. While awaiting surgery, patients should be upgraded to a more severe patient category in case of progressive symptoms.

Based on these different categories, preferred, standard, and undesirable time frames for surgical intervention are proposed (Fig. 1a, caption). In patients with no VFDs or impaired VA at diagnosis or follow-up (Group 0–1), who do not have a strict surgical indication, a surveillance policy can be a good alternative based on patient and physician preferences (shared decision-making process). If surgery is preferred, we suggest elective planning of surgery within 3 to 6 months. In Group 2, with mild VFD, we propose the following timeframes: preferred 0–2 months, standard 0–4 months, and undesirable more than 6 months. For other groups, see Fig. 1.

**Fig. 2:**
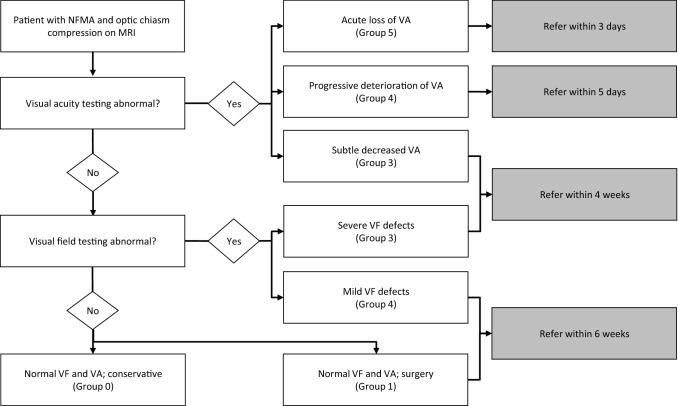
Flowchart of proposed referral delays depending on visual function and compression of optic chiasm. Proposed time(frames) for the referral of NFMA patients with visual acuity impairment or visual field defects from a non-expertise to an expertise center. *VA* visual acuity, *VF* visual field

We acknowledge that these intervals are not yet evidence-based, and thus require further future validation. However, clinical benchmarking regarding timing of surgery and establishing safety with respect to surgical delay is needed to initiate the discussion. The quality of care provided for NFMA patients that require surgical decompression of the optic chiasm is proposed to be one of the hallmarks of pituitary reference centers, such as PTCOE or European Reference Network on Rare Endocrine Conditions (Endo-ERN) [58, 65]. The assessment of quality of care should include referral and treatment delay as determining factors. The proposed clinical recommendations can aid this quality evaluation, e.g. % of patients in Group 3 operated within 2 weeks (preferred timeframe) or % of patients operated within 4 weeks (standard timeframe). In case the standard or undesirable timeframes cannot be met, suggestions for acceptable referral delays are provided in Fig. 2, which are in line with the timeframes in Fig. 1.

### Applicability of using the proposed patient stratification to assess Quality of Care

As an example, we provided data from clinical practice for clinical benchmarking in our patient cohort. Both preferred and undesirable treatment delay was seen in every patient group. Strikingly, preferred performance was best met in urgent and acute cases (Group 5), whereas standard and undesirable performance was common in severe and moderate cases (Group 3 and 4), reflecting a mismatch between desired and achieved delay in this group. In this small series, no significant adverse outcome was recorded. However, this patient stratification system needs to be validated in larger patient cohorts.

## Conclusions

The success of effective surgical decompression of the optic system in NFMA patients is very high as improvement of VFDs and VA is observed in the clear majority of patients. A practical conclusion from this systematic review is that in case of deterioration or no improvement of visual function after surgery, pituitary imaging should exclude hemorrhage. Providing uniform measures of severity of visual dysfunction from literature remains unattainable. Moreover, evidence regarding the optimal timing of surgical intervention for the individual patient is not available. However, patients with shorter treatment delay had higher postoperative recovery rates. We propose clinical recommendations to support future clinical benchmarking for the timing of surgical intervention in various categories of patients, based on objective measures of severity of VFDs and VA impairment. The proposed timelines need to be evaluated and adapted in future research projects, using international clinical networks of pituitary reference centers, such as PTCOE and Endo-ERN.

## Electronic supplementary material

Below is the link to the electronic supplementary material.

Supplementary file1 (DOCX 25 kb)

Supplementary file2 (PDF 94 kb)

Supplementary file3 (PDF 156 kb)

Supplementary file4 (DOCX 33 kb)
