# Supplementary material for: Quality of care evaluation in non-functioning pituitary adenoma with chiasm compression: visual outcomes and timing of intervention clinical recommendations based on a systematic literature review and cohort study
Source: Pituitary. 2020 May 18;23(4):417–29. doi: 10.1007/s11102-020-01044-0 (PMC7316692; doi:10.1007/s11102-020-01044-0)
Supplement: Supplementary file 1 — Supplementary file1 (DOCX 25 kb) [file 11102_2020_1044_MOESM1_ESM.docx]

**Supplementary Tables and Figures**

**Quality of care evaluation in non-functioning pituitary adenoma with chiasm compression: visual outcomes and timing of intervention
Clinical recommendations based on a systematic literature review and cohort study**

Iris C.M. Pelsma, MD, MSc * 1,4, Marco J.T. Verstegen, MD * 2,4, Friso de Vries, MD 1,4, Irene C. Notting, MD, PhD 3,4, Marike L.D. Broekman, MD, PhD2,5, Wouter R. van Furth, MD, PhD2,4, Nienke R. Biermasz, MD, PhD 1,4, Alberto M. Pereira, MD, PhD1,4

**Corresponding author**Iris C.M. Pelsma, MD, MSc
Department of Medicine, Division of Endocrinology
Leiden University Medical Center,
Albinusdreef 2,
2333 ZA Leiden, The Netherlands
Tel: +31 71 526 8172
Email: [I.C.M.Pelsma@lumc.nl](mailto:I.C.M.Pelsma@lumc.nl)

**Journal**
Pituitary

| Assessment Criteria | Qualification | Max. points |  |
| --- | --- | --- | --- |
| Age | 0 – Not described  1 – Mean (SD) or median (range) reported | 1 | |
| Pre-operative MRI | 0 – None  1 – Tumor size (width/height)  2 – Classification (e.g. HWC)  3 – Description of chiasm anatomy/signal intensity | 3 | |
| Histology | 0 – Not described  1 – Mentioned  2 – Specified (e.g. immunohistochemistry) | 2 | |
| Delta VF – Surgery | 0 – None  1 – Mean (SD) or median (range) reported  2 – Specified per patient  3 – Specified per patient & VF <1wk pre-op | 3 | |
| Pre-op VF and vision | 0 – Not described  1 – Abnormalities yes/no  2 – Specified per visual quadrant  3 – Visual field and visual acuity  4 – VF, VA and VEP/RNFL | 4 | |
| Post-op VF and vision | 0 – Not described  1 – Abnormalities yes/no  2 – Specified per visual quadrant  3 – Visual field and visual acuity  4 – VF, VA and VEP/RNFL | 4 | |
| Follow-up time | 0 – Not specified  1 – ≤ 3 months  2 – 3 months – 1 year  3 – ≥ 1 year | 3 | |
| Study classification | 0 – Selected retrospective  1 – Unselected retrospective  2 – Selected prospective  3 – Unselected prospective | 3 | |
| Complications | 0 – Not described  1 – Specified | 1 | |
| Ophthalmological comorbidity | 0 – Not described  1 – Short description  2 – Full description | 2 | |
| Post-op MRI | 0 – Not described  1 – Described  2 – Description of chiasm anatomy/signal intensity | 2 | |
| Pre-op pituitary function | 0 – Not described  1 – Mean (SD) or median (range) reported  2 – Specified per patient | 2 | |
| Post-op pituitary function | 0 – Not described  1 – Mean (SD) or median (range) reported  2 – Specified per patient | 2 | |
| Surgical technique | 0 – Not described  1 – Mean (SD) or median (range) reported  2 – Specified per patient | 2 | |
| Total maximum points |  | 34 | |

**Supplementary Table 1 Quality Assessment Tool**

All studies included for full text review were scored using this QAT. Maximum total scores were 34 points (100%). Delta VF – surgery is defined as the duration between visual field testing and surgical intervention

MRI, magnetic resonance imaging; VF, visual field; Pre-op, pre-operative; Post-op, post-operative; QAT, Quality Assessment Tool

**Supplementary Figure 1 Flowchart of article inclusion**

All articles derived from the search were screened based on title and abstract, followed by full text screening when applicable. Reasons for exclusion of articles upon full text analysis were: unspecified outcomes (N=131), defined as outcomes reported for all pituitary adenoma patients regardless of functioning status of the adenoma, other reasons (N=19), English Title and Abstract, whilst full text was written in another language and unavailability of the full text (N=65). 44 articles were included in this review.

N, number of articles

| **Quality assessment** |  |  |
| --- | --- | --- |
| **Sub scores** | Timing of surgery | 0 (0 - 0) |
|  | Pre-operative visual function tests | 1 (1 - 3) |
|  | Post-operative visual function tests | 1 (1 - 3) |
| **Total score** | Points | 135 (11.0 – 15.8) |
|  | Percentage | 39.7 (32.4 – 46.4) |

**Supplementary Table 2 Quality assessment of the included articles**

Data are shown as median (IQR) unless otherwise specified. Quality assessment scores were calculated using the QAT for all included articles (N=42). Fourteen QAT sub scores were calculated, of which 3 are shown in this Table. Maximum scores for timing of surgery, pre-operative visual function and post-operative visual function were 3, 4 and 4 points, respectively. Maximum total scores were 34 points (100%).

QAT, Quality Assessment Tool

**Supplementary Figure 2 Pre- and post-operative visual field and visual acuity assessments**

Data are shown as percentage affected patients reported per study. (A) Pre-operative visual field defects in 26 studies (2,205 patients). (B) Pre-operative visual field defects in 16 studies with postoperative assessments (1,300 patients). (C) Postoperative outcome of patients with pre-operative VFDs in these 16 studies. (D) Pre-operative visual acuity defects in 12 studies (1,354 patients). (E) Pre-operative visual acuity defects in 6 studies with postoperative assessments (662 patients). (F) Postoperative outcome of patients with VFDs in these 5 studies.

VA, visual acuity; VF, visual field; VFD, visual field defects

|  | **OD** | | | | | **OS** | | | | |
| --- | --- | --- | --- | --- | --- | --- | --- | --- | --- | --- |
| **Study ID** | **VA** | **VFI** | **MD** | **VFD** | **OCT pattern** | **VA** | **VFI** | **MD** | **VFD** | **OCT pattern** |
| 1 | 1 | 97 | -0,99 | Hemianopia | Red (central) | 1 | 95 | -1,28 | Hemianopia | Red (central) |
| 2 | 1,5 | 80 | -7,21 | Hemianopia | Yellow (superior) | 1,2 | 66 | -10,37 | Other | Red (central, temporal) |
| 3 | 1 | 98 | -4,02 | Other | Missing | 1 | 94 | -5,47 | Other | Missing |
| 4 | 1 | 99 | -0,18 | None | Missing | 1 | 99 | 0,29 | None | Missing |
| 5 | 1,2 | 96 | -3,09 | Other | Yellow (temporal) | 1 | 95 | -3,23 | Other | Green |
| 6 | 1 | 100 | 2,02 | None | Missing | 1 | 98 | -2,13 | Hemianopia | Missing |
| 7 | 1 | 98 | 0,41 | None | Groen | 1 | 99 | -0,48 | None | Missing |
| 8 | 1 | 93 | -3,92 | Other | Missing | 0,9 | 98 | -0,91 | None | Missing |
| 9 | 1 | 96 | -4,03 | None | Missing | 1 | 96 | -1,93 | None | Missing |
| 10 | 0,8 | 67 | -9,97 | Hemianopia | Red (central, nasal) | 0,9 | 46 | -18,61 | Other | Red |
| 11 | 1 | 63 | -4,25 | Other | Missing | 1 | 97 | -12,47 | None | Missing |
| 12 | 1 | 100 | 0,41 | None | Missing | 0,9 | 82 | -7,19 | Quadrantanopia | Missing |
| 13 | 1 | 94 | -2,91 | Quadrantanopia | Green | 0,7 | 67 | -9,25 | Hemianopia | Green |
| 14 | 1 | 95 | -4,91 | Other | Green | 1 | 96 | -3,61 | Other | Yellow (temporal) |
| 15 | 0,7 | 92 | -3,97 | Hemianopia | Missing | 1 | 95 | -4,44 | Other | Missing |
| 16 | 1,2 | 94 | -3,73 | Quadrantanopia | Missing | 1,2 | 98 | -3,34 | Other | Missing |
| 17 | 0,9 | 67 | -11,69 | Hemianopia | Missing | 0,2 | 43 | -19,1 | Hemianopia | Missing |
| 18 | 1,2 | 82 | -6,09 | Quadrantanopia | Yellow (central) | 1 | 69 | -8,03 | Hemianopia | Red (central, nasal) |
| 19 | 0,3 | 88 | -5,04 | Hemianopia | Red (central) | 0,6 | 78 | -7,3 | Hemianopia | Missing |
| 20 | 1 | 88 | -7,61 | Hemianopia | Red (superior) | 1 | 97 | -5,72 | Hemianopia | Red (superior) |
| 21 | 1 | 91 | -6,04 | Other | Missing | 1 | 94 | -3,95 | Other | Missing |
| 22 | 1 | 86 | -5,69 | Quadrantanopia | Yellow (nasal) | 0,1 | 59 | -12,4 | Hemianopia | Green |
| 23 | 0,8 | 89 | -4,54 | Hemianopia | Yellow (nasal) | 1 | 92 | -2,6 | Hemianopia | Red (nasal) |
| 24 | 0,6 | 41 | -19,97 | Hemianopia | Green | 0,1 | 32 | -20,92 | Hemianopia | Red (temporal) |
| 25 | 1 | 59 | -18,73 | Other | Green | 0,8 | 52 | -18,9 | Other | Green |
| 26 | 1 | 85 | -8,97 | Quadrantanopia | Missing | 1 | 84 | -5,74 | Hemianopia | Missing |
| 27 | 0,7 | 83 | -5,66 | Quadrantanopia | Missing | 0,8 | 95 | -3,48 | None | Missing |
| 28 | 0,6 | 98 | -2,83 | Hemianopia | Green | 1 | 79 | -10,74 | Other | Yellow (nasal) |
| 29 | 0,9 | 68 | -10,18 | Hemianopia | Missing | 0,4 | 42 | -18,28 | Hemianopia | Missing |
| 30 | 1 | 25 | -24,11 | Other | Red (central) | 0,3 | 2 | -30,52 | Blind | Red (central) |

**Supplementary Table 3 Visual characteristics of the cohort of NFMA patients**

For all included 30 patients, outcomes of the preoperative visual function tests, including visual field and acuity assessments, are shown for each eye. OCTs were performed in 16 patients.

MD, mean deviation; OD, right eye; OS, left eye; VA, visual acuity; VFI, visual field index; VFD, visual field defects
