# Supplementary figures and images for: Quality of care evaluation in non-functioning pituitary adenoma with chiasm compression: visual outcomes and timing of intervention clinical recommendations based on a systematic literature review and cohort study
Source: Pituitary. 2020 May 18;23(4):417–29. doi: 10.1007/s11102-020-01044-0 (PMC7316692; doi:10.1007/s11102-020-01044-0)

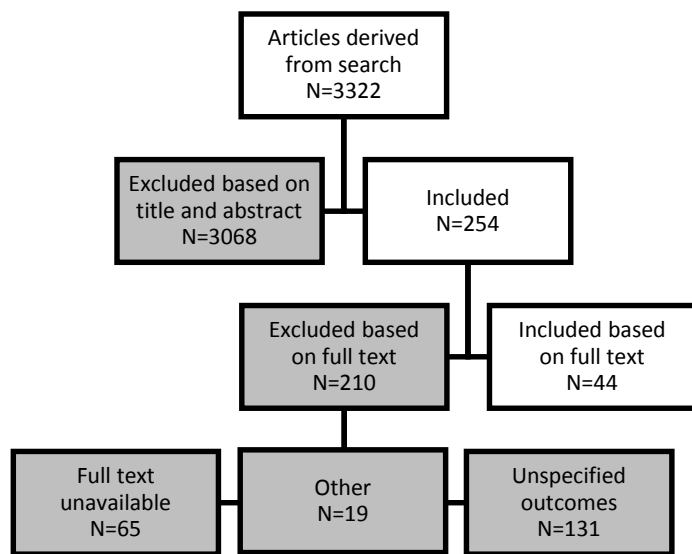

Supplement: Supplementary file 2 — Supplementary file2 (PDF 94 kb) [file 11102_2020_1044_MOESM2_ESM.pdf]

**A**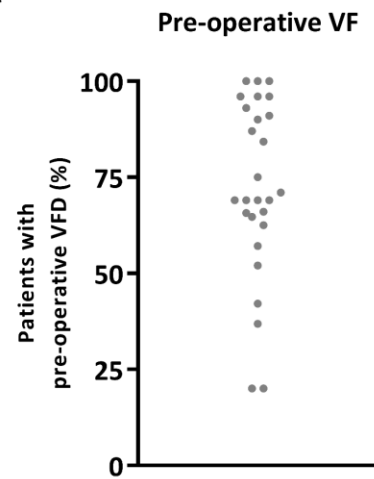**B**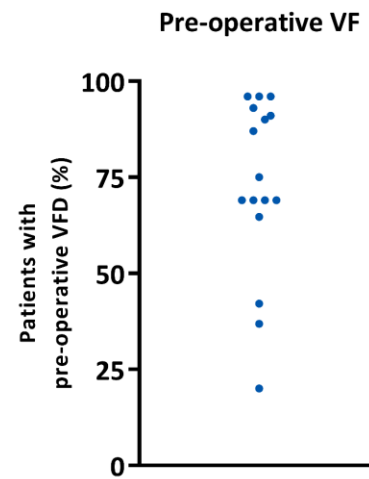**C**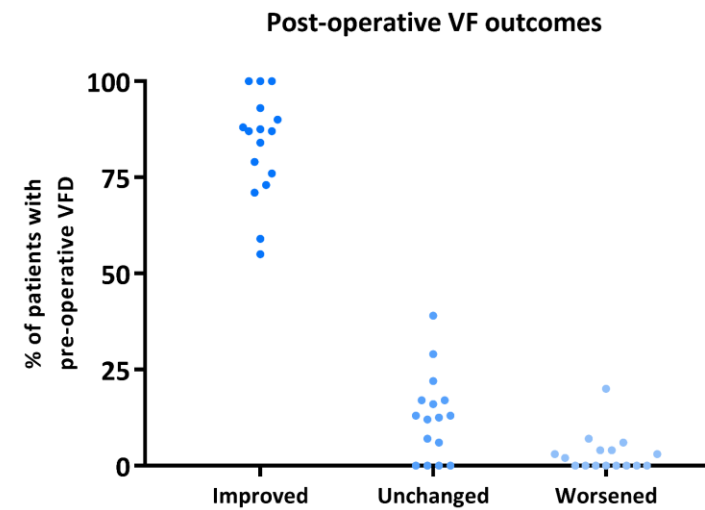**D**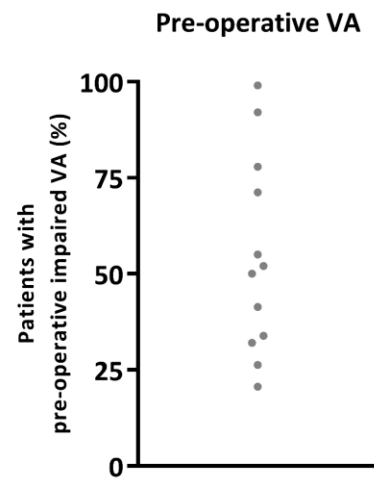**E**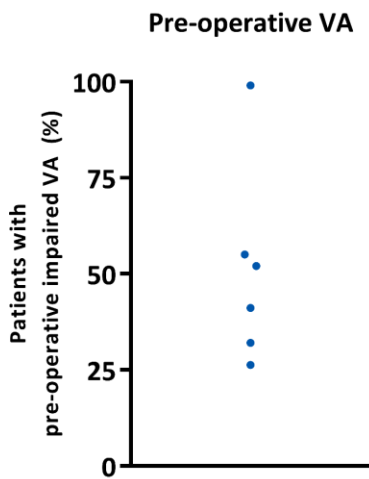**F**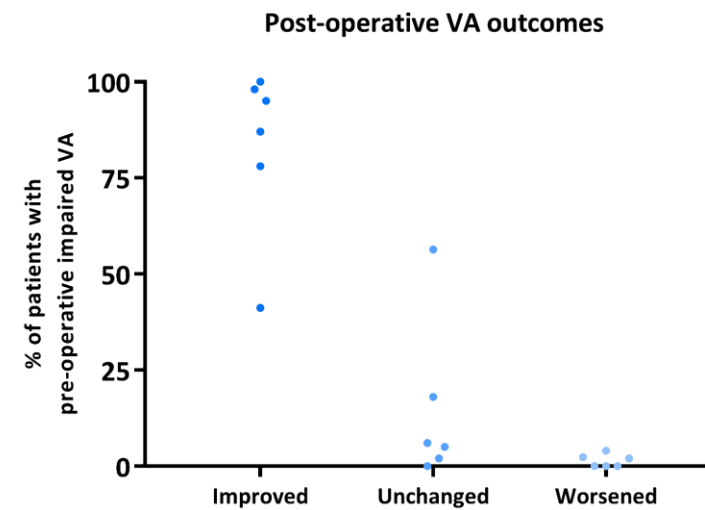

Supplement: Supplementary file 3 — Supplementary file3 (PDF 156 kb) [file 11102_2020_1044_MOESM3_ESM.pdf]
