# Supplementary material for: Quality of care evaluation in non-functioning pituitary adenoma with chiasm compression: visual outcomes and timing of intervention clinical recommendations based on a systematic literature review and cohort study
Source: Pituitary. 2020 May 18;23(4):417–29. doi: 10.1007/s11102-020-01044-0 (PMC7316692; doi:10.1007/s11102-020-01044-0)
Supplement: Supplementary file 4 — Supplementary file4 (DOCX 33 kb) [file 11102_2020_1044_MOESM4_ESM.docx]

**Supplementary File I Search strategy**

Databases:

**PubMed**

<http://www.ncbi.nlm.nih.gov/entrez/query.fcgi?otool=leiden>

((((("transsphenoidal surgery"[tw] OR "transcranial surgery"[tw] OR "Surgical Procedures, Operative"[mesh] **OR operati*[tw] OR operate*[tw] OR "surgery"[tw]**) AND ("pituitary adenomas"[tw] OR "pituitary adenoma"[tw] OR "pituitary macroadenomas"[tw] OR "pituitary macroadenoma"[tw] OR "pituitary macro-adenomas"[tw] OR "pituitary macro-adenoma"[tw] OR "Pituitary Neoplasms"[mesh] OR "non functioning pituitary adenomas"[tw] OR "non functioning pituitary adenoma"[tw] OR "Non-functioning pituitary tumours"[tw] OR "Non-functioning pituitary tumour"[tw] OR "Non-functioning pituitary tumors"[tw] OR "Non-functioning pituitary tumor"[tw] OR "nonfunctioning pituitary adenomas"[tw] OR "nonfunctioning pituitary adenoma"[tw] OR "Nonfunctioning pituitary tumours"[tw] OR "Nonfunctioning pituitary tumour"[tw] OR "Nonfunctioning pituitary tumors"[tw] OR "Nonfunctioning pituitary tumor"[tw] OR "nonfunctioning pituitary"[tw] OR "non functioning pituitary"[tw] OR (("pituitary adenoma"[tw] OR "pituitary adenomas"[tw] OR "pituitary macroadenoma"[tw] OR "pituitary macroadenomas"[tw] OR "pituitary macro-adenoma"[tw] OR "pituitary macro-adenomas"[tw] OR "pituitary tumor"[tw] OR "pituitary tumors"[tw] OR "pituitary tumour"[tw] OR "pituitary tumours"[tw] OR "pituitary neoplasms"[tw]) AND ("non functioning"[tw] OR "nonfunctioning"[tw] OR non-function*[tw] OR nonfunction*[tw])))) OR "Pituitary Neoplasms/surgery"[mesh]) AND ("Vision, Ocular"[mesh] OR "vision"[tw] OR "visual acuity"[tw] OR "visual fields"[tw] OR "visual field recovery"[tw] OR "visual field"[tw] OR "visual disturbance"[tw] OR "visual disturbances"[tw] OR "VF assessment"[tw] OR "VF recovery"[tw] OR ("VF"[tiab] AND "eyes"[tiab]) OR "visual acuity"[tw] OR "VF deficit"[tw] OR "Vision Disorders"[mesh] OR "Visual Fields"[mesh] OR "Humphrey Field Analyzer"[tw] OR "visual outcome"[tw] OR "visual outcomes"[tw] OR "Visual Acuity"[mesh] OR "Visual parameters"[tw] OR "Visual parameter"[tw] OR "visual prognosis"[tw] OR "vision outcome"[tw] OR "vision outcomes"[tw] OR "Vision parameters"[tw] OR "Vision parameter"[tw] OR "vision prognosis"[tw] **OR visual function*[tw] OR visual loss*[tw]**) AND ("Pre-existing"[tw] OR "preexisting"[tw] OR "postoperatively"[tw] OR **post-operati*[tw] OR postoperati*[tw]** OR "time course"[tw] OR "predictive factors"[tw] OR "predictive factor"[tw] OR "early intervention"[tw] OR "Predictive Value of Tests"[mesh] OR "before and after"[tw] OR "timing"[tw] OR "Time Factors"[mesh] OR "years after"[tw] OR "year after"[tw] OR "months after"[tw] OR "month after"[tw] OR "weeks after"[tw] OR "week after"[tw] OR "days after"[tw] OR "day after"[tw] OR "before and after"[tw] **OR "natural course"[tw]**)) OR (("pituitary adenomas"[tw] OR "pituitary adenoma"[tw] OR "pituitary macroadenomas"[tw] OR "pituitary macroadenoma"[tw] OR "pituitary macro-adenomas"[tw] OR "pituitary macro-adenoma"[tw] OR "Pituitary Neoplasms"[mesh] OR "non functioning pituitary adenomas"[tw] OR "non functioning pituitary adenoma"[tw] OR "Non-functioning pituitary tumours"[tw] OR "Non-functioning pituitary tumour"[tw] OR "Non-functioning pituitary tumors"[tw] OR "Non-functioning pituitary tumor"[tw] OR "nonfunctioning pituitary adenomas"[tw] OR "nonfunctioning pituitary adenoma"[tw] OR "Nonfunctioning pituitary tumours"[tw] OR "Nonfunctioning pituitary tumour"[tw] OR "Nonfunctioning pituitary tumors"[tw] OR "Nonfunctioning pituitary tumor"[tw] OR "nonfunctioning pituitary"[tw] OR "non functioning pituitary"[tw] OR (("pituitary adenoma"[tw] OR "pituitary adenomas"[tw] OR "pituitary macroadenoma"[tw] OR "pituitary macroadenomas"[tw] OR "pituitary macro-adenoma"[tw] OR "pituitary macro-adenomas"[tw] OR "pituitary tumor"[tw] OR "pituitary tumors"[tw] OR "pituitary tumour"[tw] OR "pituitary tumours"[tw] OR "pituitary neoplasms"[tw]) AND ("non functioning"[tw] OR "nonfunctioning"[tw] OR non-function*[tw] OR nonfunction*[tw]))) AND ("timing"[tw] OR "Time Factors"[mesh] OR "years after"[tw] OR "year after"[tw] OR "months after"[tw] OR "month after"[tw] OR "weeks after"[tw] OR "week after"[tw] OR "days after"[tw] OR "day after"[tw] OR "before and after"[tw]) AND ("surgery"[subheading] OR "surgery"[tw] OR "Surgical Procedures, Operative"[mesh] **OR operati*[tw] OR operate*[tw] OR "surgery"[tw]**)) OR (("pituitary adenomas"[tw] OR "pituitary adenoma"[tw] OR "pituitary macroadenomas"[tw] OR "pituitary macroadenoma"[tw] OR "pituitary macro-adenomas"[tw] OR "pituitary macro-adenoma"[tw] OR "Pituitary Neoplasms"[mesh] OR "non functioning pituitary adenomas"[tw] OR "non functioning pituitary adenoma"[tw] OR "Non-functioning pituitary tumours"[tw] OR "Non-functioning pituitary tumour"[tw] OR "Non-functioning pituitary tumors"[tw] OR "Non-functioning pituitary tumor"[tw] OR "nonfunctioning pituitary adenomas"[tw] OR "nonfunctioning pituitary adenoma"[tw] OR "Nonfunctioning pituitary tumours"[tw] OR "Nonfunctioning pituitary tumour"[tw] OR "Nonfunctioning pituitary tumors"[tw] OR "Nonfunctioning pituitary tumor"[tw] OR "nonfunctioning pituitary"[tw] OR "non functioning pituitary"[tw] OR (("pituitary adenoma"[tw] OR "pituitary adenomas"[tw] OR "pituitary macroadenoma"[tw] OR "pituitary macroadenomas"[tw] OR "pituitary macro-adenoma"[tw] OR "pituitary macro-adenomas"[tw] OR "pituitary tumor"[tw] OR "pituitary tumors"[tw] OR "pituitary tumour"[tw] OR "pituitary tumours"[tw] OR "pituitary neoplasms"[tw]) AND ("non functioning"[tw] OR "nonfunctioning"[tw] OR non-function*[tw] OR nonfunction*[tw])) OR "Pituitary Neoplasms"[mesh] OR "Pituitary tumor"[tw] OR "Pituitary tumors"[tw] OR "Pituitary tumour"[tw] OR "Pituitary tumours"[tw] OR "Pituitary adenoma"[tw] OR "Pituitary adenomas"[tw] OR "sellar tumor"[tw] OR "sellar tumors"[tw] OR "sellar tumour"[tw] OR "sellar tumours"[tw] OR "sellar neoplasm"[tw] OR "sellar neoplasms"[tw] OR ("sellar"[ti] AND "adenoma"[ti]) OR ("sellar"[ti] AND "adenomas"[ti]) OR "suprasellar tumor"[tw] OR "suprasellar tumors"[tw] OR "suprasellar tumour"[tw] OR "suprasellar tumours"[tw] OR "suprasellar neoplasm"[tw] OR "suprasellar neoplasms"[tw] OR ("suprasellar"[ti] AND "adenoma"[ti]) OR "suprasellar adenomas"[tw] OR "pituitary gland adenoma"[tw] OR "pituitary gland adenomas"[tw] OR "pituitary gland tumor"[tw] OR "pituitary gland tumors"[tw] OR "pituitary gland tumour"[tw] OR "pituitary gland tumours"[tw] OR "pituitary gland neoplasm"[tw] OR "pituitary gland neoplasms"[tw]) AND ("timing"[tw] OR "Time Factors"[mesh] OR "years after"[tw] OR "year after"[tw] OR "months after"[tw] OR "month after"[tw] OR "weeks after"[tw] OR "week after"[tw] OR "days after"[tw] OR "day after"[tw] OR "before and after"[tw]) AND ("surgery"[subheading] OR "surgery"[tw] OR "Surgical Procedures, Operative"[mesh] **OR operati*[tw] OR operate*[tw] OR "surgery"[tw]**)) OR (("pituitary adenomas"[ti] OR "pituitary adenoma"[ti] OR "pituitary macroadenomas"[ti] OR "pituitary macroadenoma"[ti] OR "pituitary macro-adenomas"[ti] OR "pituitary macro-adenoma"[ti] OR "Pituitary Neoplasms"[majr] OR "non functioning pituitary adenomas"[ti] OR "non functioning pituitary adenoma"[ti] OR "Non-functioning pituitary tumours"[ti] OR "Non-functioning pituitary tumour"[ti] OR "Non-functioning pituitary tumors"[ti] OR "Non-functioning pituitary tumor"[ti] OR "nonfunctioning pituitary adenomas"[ti] OR "nonfunctioning pituitary adenoma"[ti] OR "Nonfunctioning pituitary tumours"[ti] OR "Nonfunctioning pituitary tumour"[ti] OR "Nonfunctioning pituitary tumors"[ti] OR "Nonfunctioning pituitary tumor"[ti] OR "nonfunctioning pituitary"[ti] OR "non functioning pituitary"[ti] OR (("pituitary adenoma"[ti] OR "pituitary adenomas"[ti] OR "pituitary macroadenoma"[ti] OR "pituitary macroadenomas"[ti] OR "pituitary macro-adenoma"[ti] OR "pituitary macro-adenomas"[ti] OR "pituitary tumor"[ti] OR "pituitary tumors"[ti] OR "pituitary tumour"[ti] OR "pituitary tumours"[ti] OR "pituitary neoplasms"[ti]) AND ("non functioning"[ti] OR "nonfunctioning"[ti] OR non-function*[ti] OR nonfunction*[ti]))) AND ("timing"[tw] OR "Time Factors"[mesh] OR "years after"[tw] OR "year after"[tw] OR "months after"[tw] OR "month after"[tw] OR "weeks after"[tw] OR "week after"[tw] OR "days after"[tw] OR "day after"[tw] OR "before and after"[tw] **OR "time"[tw]**) AND ("surgery"[tiab] OR "Surgical Procedures, Operative"[majr])) OR (((("transsphenoidal surgery"[tw] OR "transcranial surgery"[tw] OR "Surgical Procedures, Operative"[mesh] **OR operati*[tw] OR operate*[tw] OR "surgery"[tw]**) AND ("pituitary adenomas"[tw] OR "pituitary adenoma"[tw] OR "pituitary macroadenomas"[tw] OR "pituitary macroadenoma"[tw] OR "pituitary macro-adenomas"[tw] OR "pituitary macro-adenoma"[tw] OR "Pituitary Neoplasms"[mesh] OR "non functioning pituitary adenomas"[tw] OR "non functioning pituitary adenoma"[tw] OR "Non-functioning pituitary tumours"[tw] OR "Non-functioning pituitary tumour"[tw] OR "Non-functioning pituitary tumors"[tw] OR "Non-functioning pituitary tumor"[tw] OR "nonfunctioning pituitary adenomas"[tw] OR "nonfunctioning pituitary adenoma"[tw] OR "Nonfunctioning pituitary tumours"[tw] OR "Nonfunctioning pituitary tumour"[tw] OR "Nonfunctioning pituitary tumors"[tw] OR "Nonfunctioning pituitary tumor"[tw] OR "nonfunctioning pituitary"[tw] OR "non functioning pituitary"[tw] OR (("pituitary adenoma"[tw] OR "pituitary adenomas"[tw] OR "pituitary macroadenoma"[tw] OR "pituitary macroadenomas"[tw] OR "pituitary macro-adenoma"[tw] OR "pituitary macro-adenomas"[tw] OR "pituitary tumor"[tw] OR "pituitary tumors"[tw] OR "pituitary tumour"[tw] OR "pituitary tumours"[tw] OR "pituitary neoplasms"[tw]) AND ("non functioning"[tw] OR "nonfunctioning"[tw] OR non-function*[tw] OR nonfunction*[tw])))) OR "Pituitary Neoplasms/surgery"[mesh]) AND ("Vision, Ocular"[mesh] OR "vision"[tw] OR "visual acuity"[tw] OR "visual fields"[tw] OR "visual field recovery"[tw] OR "visual field"[tw] OR "visual disturbance"[tw] OR "visual disturbances"[tw] OR "VF assessment"[tw] OR "VF recovery"[tw] OR ("VF"[tw] AND "eyes"[tw]) OR "visual acuity"[tw] OR "VF deficit"[tw] OR "Vision Disorders"[mesh] OR "Visual Fields"[mesh] OR "Humphrey Field Analyzer"[tw] OR "visual outcome"[tw] OR "visual outcomes"[tw] OR "Visual Acuity"[mesh] OR "Visual parameters"[tw] OR "Visual parameter"[tw] OR "visual prognosis"[tw] OR "vision outcome"[tw] OR "vision outcomes"[tw] OR "Vision parameters"[tw] OR "Vision parameter"[tw] OR "vision prognosis"[tw] **OR visual function*[tw] OR visual loss*[tw]**) AND ("follow-up"[tw] OR followup*[tw] OR "Follow-Up Studies"[mesh]) AND ("timing"[tw] OR "Time Factors"[mesh] OR "years after"[tw] OR "year after"[tw] OR "months after"[tw] OR "month after"[tw] OR "weeks after"[tw] OR "week after"[tw] OR "days after"[tw] OR "day after"[tw] OR "before and after"[tw] **OR "time"[tw]**))) **NOT ("Animals"[mesh] NOT "Humans"[mesh]) NOT ("Letter"[Publication Type] NOT "Clinical Study"[Publication Type])**

**EMBASE**

<http://gateway.ovid.com/ovidweb.cgi?T=JS&MODE=ovid&NEWS=N&PAGE=main&D=emez>

((((((transsphenoidal surgery/ OR "transsphenoidal surgery".mp OR "transcranial surgery".mp **OR operat*.mp OR "surgery".mp**) AND (hypophysis adenoma/ OR (pituitary adenomas OR pituitary adenoma OR pituitary macroadenomas OR pituitary macroadenoma).mp OR exp hypophysis tumor/ OR ("non functioning pituitary adenomas" OR "non functioning pituitary adenoma" OR "Non-functioning pituitary tumours" OR "Non-functioning pituitary tumour" OR "Non-functioning pituitary tumors" OR "Non-functioning pituitary tumor" OR "nonfunctioning pituitary adenomas" OR "nonfunctioning pituitary adenoma" OR "Nonfunctioning pituitary tumours" OR "Nonfunctioning pituitary tumour" OR "Nonfunctioning pituitary tumors" OR "Nonfunctioning pituitary tumor" OR "nonfunctioning pituitary" OR "non functioning pituitary").mp OR ((pituitary adenoma OR pituitary adenomas OR pituitary macroadenoma OR pituitary macroadenomas OR pituitary macro-adenoma OR pituitary macro-adenomas OR pituitary tumor OR pituitary tumors OR pituitary tumour OR pituitary tumours OR pituitary neoplasms) AND ("non functioning" OR nonfunctioning)).mp)) OR exp hypophysis tumor/su) AND (exp vision/ OR exp visual system parameters/ OR ("visual acuity" OR "visual fields" OR "visual field recovery" OR "visual field" OR "visual disturbance" OR "visual disturbances" OR "VF assessment" OR "VF recovery" OR (VF AND eyes) OR "visual acuity" OR "VF deficit").mp OR exp visual disorder/ OR ("Humphrey Field Analyzer" OR "visual outcome" OR "Visual parameters" OR "Visual parameter" OR "visual prognosis" OR vision **OR visual function* OR visual loss***).mp) AND ((Pre-existing OR postoperatively OR post-operatively OR "time course" OR "predictive factors" OR "predictive factor" OR "early intervention").mp OR exp prediction/ OR exp predictive value/ OR "before and after".mp OR timing.mp OR exp time/ **OR ("years after" OR "year after" OR "months after" OR "month after" OR "weeks after" OR "week after" OR "days after" OR "day after" OR "before and after").mp**))) OR ((("non functioning pituitary adenomas" OR "non functioning pituitary adenoma" OR "Non-functioning pituitary tumours" OR "Non-functioning pituitary tumour" OR "Non-functioning pituitary tumors" OR "Non-functioning pituitary tumor" OR "nonfunctioning pituitary adenomas" OR "nonfunctioning pituitary adenoma" OR "Nonfunctioning pituitary tumours" OR "Nonfunctioning pituitary tumour" OR "Nonfunctioning pituitary tumors" OR "Nonfunctioning pituitary tumor" OR "nonfunctioning pituitary" OR "non functioning pituitary" OR ((pituitary adenoma OR pituitary adenomas OR pituitary macroadenoma OR pituitary macroadenomas OR pituitary macro-adenoma OR pituitary macro-adenomas OR pituitary tumor OR pituitary tumors OR pituitary tumour OR pituitary tumours OR pituitary neoplasms) AND ("non functioning" OR nonfunctioning)).mp) AND (timing.mp OR exp time/ **OR ("years after" OR "year after" OR "months after" OR "month after" OR "weeks after" OR "week after" OR "days after" OR "day after" OR "before and after").mp**) AND (surgery.mp OR surgical*.mp OR surgeon*.mp OR operat*.mp OR exp surgery/))) OR (((("non functioning pituitary adenomas" OR "non functioning pituitary adenoma" OR "Non-functioning pituitary tumours" OR "Non-functioning pituitary tumour" OR "Non-functioning pituitary tumors" OR "Non-functioning pituitary tumor" OR "nonfunctioning pituitary adenomas" OR "nonfunctioning pituitary adenoma" OR "Nonfunctioning pituitary tumours" OR "Nonfunctioning pituitary tumour" OR "Nonfunctioning pituitary tumors" OR "Nonfunctioning pituitary tumor" OR "nonfunctioning pituitary" OR "non functioning pituitary" OR ((pituitary adenoma OR pituitary adenomas OR pituitary macroadenoma OR pituitary macroadenomas OR pituitary macro-adenoma OR pituitary macro-adenomas OR pituitary tumor OR pituitary tumors OR pituitary tumour OR pituitary tumours OR pituitary neoplasms) AND ("non functioning" OR nonfunctioning))).mp OR exp hypophysis tumor/ OR ("Pituitary tumor" OR "Pituitary tumors" OR "Pituitary tumour" OR "Pituitary tumours" OR "Pituitary adenoma" OR "Pituitary adenomas" OR "sellar tumor" OR "sellar tumors" OR "sellar tumour" OR "sellar tumours" OR "sellar neoplasm" OR "sellar neoplasms").mp OR (sellar.ti AND adenoma.ti) OR (sellar.ti AND adenomas.ti) OR ("suprasellar tumor" OR "suprasellar tumors" OR "suprasellar tumour" OR "suprasellar tumours" OR "suprasellar neoplasm" OR "suprasellar neoplasms").mp OR (suprasellar.ti AND adenoma.ti) OR ("suprasellar adenomas" OR "pituitary gland adenoma" OR "pituitary gland adenomas" OR "pituitary gland tumor" OR "pituitary gland tumors" OR "pituitary gland tumour" OR "pituitary gland tumours" OR "pituitary gland neoplasm" OR "pituitary gland neoplasms").mp) AND (timing.mp OR exp time/ OR (**"years after" OR "year after" OR "months after" OR "month after" OR "weeks after" OR "week after" OR "days after" OR "day after" OR "before and after").mp**) AND (surgery.mp OR surgical*.mp OR surgeon*.mp OR exp surgery/ OR operat*.mp))) OR (((((transsphenoidal surgery/ OR "transsphenoidal surgery".mp OR "transcranial surgery".mp **OR operat*.mp OR "surgery".mp**) AND (hypophysis adenoma/ OR (pituitary adenomas OR pituitary adenoma OR pituitary macroadenomas OR pituitary macroadenoma).mp OR exp hypophysis tumor/ OR ("non functioning pituitary adenomas" OR "non functioning pituitary adenoma" OR "Non-functioning pituitary tumours" OR "Non-functioning pituitary tumour" OR "Non-functioning pituitary tumors" OR "Non-functioning pituitary tumor" OR "nonfunctioning pituitary adenomas" OR "nonfunctioning pituitary adenoma" OR "Nonfunctioning pituitary tumours" OR "Nonfunctioning pituitary tumour" OR "Nonfunctioning pituitary tumors" OR "Nonfunctioning pituitary tumor" OR "nonfunctioning pituitary" OR "non functioning pituitary").mp OR ((pituitary adenoma OR pituitary adenomas OR pituitary macroadenoma OR pituitary macroadenomas OR pituitary macro-adenoma OR pituitary macro-adenomas OR pituitary tumor OR pituitary tumors OR pituitary tumour OR pituitary tumours OR pituitary neoplasms) AND ("non functioning" OR nonfunctioning)).mp)) OR exp hypophysis tumor/su) AND (exp vision/ OR exp visual system parameters/ OR ("visual acuity" OR "visual fields" OR "visual field recovery" OR "visual field" OR "visual disturbance" OR "visual disturbances" OR "VF assessment" OR "VF recovery" OR (VF AND eyes) OR "visual acuity" OR "VF deficit").mp OR exp visual disorder/ OR ("Humphrey Field Analyzer" OR "visual outcome" OR "Visual parameters" OR "Visual parameter" OR "visual prognosis" OR vision **OR visual function* OR visual loss***).mp) AND ("follow-up".mp OR followup*.mp OR "Follow-Up"/) AND ("timing".mp OR exp "Time"/ OR "years after".mp OR "year after".mp OR "months after".mp OR "month after".mp OR "weeks after".mp OR "week after".mp OR "days after".mp OR "day after".mp OR "before and after".mp **OR "time".mp**)))) **AND exp "Humans"/ NOT ("Letter"/ NOT exp "Clinical Study"/) NOT conference review.pt**

**exp Case Report/**

**limit to conference abstract**

**Web of Science**

<http://isiknowledge.com/wos>

TS=((((((transsphenoidal surgery OR "transsphenoidal surgery" OR "transcranial surgery" **OR operat* OR "surgery"**) AND (hypophysis adenoma OR (pituitary adenomas OR pituitary adenoma OR pituitary macroadenomas OR pituitary macroadenoma) OR hypophysis tumor OR ("non functioning pituitary adenomas" OR "non functioning pituitary adenoma" OR "Non-functioning pituitary tumours" OR "Non-functioning pituitary tumour" OR "Non-functioning pituitary tumors" OR "Non-functioning pituitary tumor" OR "nonfunctioning pituitary adenomas" OR "nonfunctioning pituitary adenoma" OR "Nonfunctioning pituitary tumours" OR "Nonfunctioning pituitary tumour" OR "Nonfunctioning pituitary tumors" OR "Nonfunctioning pituitary tumor" OR "nonfunctioning pituitary" OR "non functioning pituitary") OR ((pituitary adenoma OR pituitary adenomas OR pituitary macroadenoma OR pituitary macroadenomas OR pituitary macro-adenoma OR pituitary macro-adenomas OR pituitary tumor OR pituitary tumors OR pituitary tumour OR pituitary tumours OR pituitary neoplasms) AND ("non functioning" OR nonfunctioning))))) AND (vision OR visual system parameters OR ("visual acuity" OR "visual fields" OR "visual field recovery" OR "visual field" OR "visual disturbance" OR "visual disturbances" OR "VF assessment" OR "VF recovery" OR (VF AND eyes) OR "visual acuity" OR "VF deficit") OR visual disorder OR ("Humphrey Field Analyzer" OR "visual outcome" OR "Visual parameters" OR "Visual parameter" OR "visual prognosis" OR vision **OR visual function* OR visual loss***)) AND ((Pre-existing OR postoperatively OR post-operatively OR "time course" OR "predictive factors" OR "predictive factor" OR "early intervention") OR prediction OR predictive value OR "before and after" OR timing OR time **OR ("years after" OR "year after" OR "months after" OR "month after" OR "weeks after" OR "week after" OR "days after" OR "day after" OR "before and after")**))) OR ((("non functioning pituitary adenomas" OR "non functioning pituitary adenoma" OR "Non-functioning pituitary tumours" OR "Non-functioning pituitary tumour" OR "Non-functioning pituitary tumors" OR "Non-functioning pituitary tumor" OR "nonfunctioning pituitary adenomas" OR "nonfunctioning pituitary adenoma" OR "Nonfunctioning pituitary tumours" OR "Nonfunctioning pituitary tumour" OR "Nonfunctioning pituitary tumors" OR "Nonfunctioning pituitary tumor" OR "nonfunctioning pituitary" OR "non functioning pituitary" OR ((pituitary adenoma OR pituitary adenomas OR pituitary macroadenoma OR pituitary macroadenomas OR pituitary macro-adenoma OR pituitary macro-adenomas OR pituitary tumor OR pituitary tumors OR pituitary tumour OR pituitary tumours OR pituitary neoplasms) AND ("non functioning" OR nonfunctioning))) AND (timing OR time **OR ("years after" OR "year after" OR "months after" OR "month after" OR "weeks after" OR "week after" OR "days after" OR "day after" OR "before and after")**) AND (surgery OR surgical* OR surgeon* OR operat* OR surgery))) OR (((("non functioning pituitary adenomas" OR "non functioning pituitary adenoma" OR "Non-functioning pituitary tumours" OR "Non-functioning pituitary tumour" OR "Non-functioning pituitary tumors" OR "Non-functioning pituitary tumor" OR "nonfunctioning pituitary adenomas" OR "nonfunctioning pituitary adenoma" OR "Nonfunctioning pituitary tumours" OR "Nonfunctioning pituitary tumour" OR "Nonfunctioning pituitary tumors" OR "Nonfunctioning pituitary tumor" OR "nonfunctioning pituitary" OR "non functioning pituitary" OR ((pituitary adenoma OR pituitary adenomas OR pituitary macroadenoma OR pituitary macroadenomas OR pituitary macro-adenoma OR pituitary macro-adenomas OR pituitary tumor OR pituitary tumors OR pituitary tumour OR pituitary tumours OR pituitary neoplasms) AND ("non functioning" OR nonfunctioning))) OR hypophysis tumor OR ("Pituitary tumor" OR "Pituitary tumors" OR "Pituitary tumour" OR "Pituitary tumours" OR "Pituitary adenoma" OR "Pituitary adenomas" OR "sellar tumor" OR "sellar tumors" OR "sellar tumour" OR "sellar tumours" OR "sellar neoplasm" OR "sellar neoplasms") OR (sellar.ti AND adenoma.ti) OR (sellar.ti AND adenomas.ti) OR ("suprasellar tumor" OR "suprasellar tumors" OR "suprasellar tumour" OR "suprasellar tumours" OR "suprasellar neoplasm" OR "suprasellar neoplasms") OR (suprasellar.ti AND adenoma.ti) OR ("suprasellar adenomas" OR "pituitary gland adenoma" OR "pituitary gland adenomas" OR "pituitary gland tumor" OR "pituitary gland tumors" OR "pituitary gland tumour" OR "pituitary gland tumours" OR "pituitary gland neoplasm" OR "pituitary gland neoplasms")) AND (timing OR time OR (**"years after" OR "year after" OR "months after" OR "month after" OR "weeks after" OR "week after" OR "days after" OR "day after" OR "before and after")**) AND (surgery OR surgical* OR surgeon* OR surgery OR operat*))) OR (((((transsphenoidal surgery OR "transsphenoidal surgery" OR "transcranial surgery" **OR operat* OR "surgery"**) AND (hypophysis adenoma OR (pituitary adenomas OR pituitary adenoma OR pituitary macroadenomas OR pituitary macroadenoma) OR hypophysis tumor OR ("non functioning pituitary adenomas" OR "non functioning pituitary adenoma" OR "Non-functioning pituitary tumours" OR "Non-functioning pituitary tumour" OR "Non-functioning pituitary tumors" OR "Non-functioning pituitary tumor" OR "nonfunctioning pituitary adenomas" OR "nonfunctioning pituitary adenoma" OR "Nonfunctioning pituitary tumours" OR "Nonfunctioning pituitary tumour" OR "Nonfunctioning pituitary tumors" OR "Nonfunctioning pituitary tumor" OR "nonfunctioning pituitary" OR "non functioning pituitary") OR ((pituitary adenoma OR pituitary adenomas OR pituitary macroadenoma OR pituitary macroadenomas OR pituitary macro-adenoma OR pituitary macro-adenomas OR pituitary tumor OR pituitary tumors OR pituitary tumour OR pituitary tumours OR pituitary neoplasms) AND ("non functioning" OR nonfunctioning))))) AND (vision OR visual system parameters OR ("visual acuity" OR "visual fields" OR "visual field recovery" OR "visual field" OR "visual disturbance" OR "visual disturbances" OR "VF assessment" OR "VF recovery" OR (VF AND eyes) OR "visual acuity" OR "VF deficit") OR visual disorder OR ("Humphrey Field Analyzer" OR "visual outcome" OR "Visual parameters" OR "Visual parameter" OR "visual prognosis" OR vision **OR visual function* OR visual loss***)) AND ("follow-up" OR followup* OR "Follow-Up") AND ("timing" OR "Time" OR "years after" OR "year after" OR "months after" OR "month after" OR "weeks after" OR "week after" OR "days after" OR "day after" OR "before and after" **OR "time"**)))) **NOT ti=(animal* OR "rat" OR "rats" OR "mice" OR "mouse" OR "dog" OR "dogs" OR "canine" OR "porcine" OR "pig" OR "pigs")**

**NOT "Letter"**

**NOT TI=("Case Report" OR ("case" AND "report"))**

**limit to conference abstract**

**Cochrane**

<http://www3.interscience.wiley.com/cgi-bin/mrwhome/106568753/HOME>

ti/su/ab/kw

((((((transsphenoidal surgery OR "transsphenoidal surgery" OR "transcranial surgery" **OR operat* OR "surgery"**) AND (hypophysis adenoma OR (pituitary adenomas OR pituitary adenoma OR pituitary macroadenomas OR pituitary macroadenoma) OR hypophysis tumor OR ("non functioning pituitary adenomas" OR "non functioning pituitary adenoma" OR "Non-functioning pituitary tumours" OR "Non-functioning pituitary tumour" OR "Non-functioning pituitary tumors" OR "Non-functioning pituitary tumor" OR "nonfunctioning pituitary adenomas" OR "nonfunctioning pituitary adenoma" OR "Nonfunctioning pituitary tumours" OR "Nonfunctioning pituitary tumour" OR "Nonfunctioning pituitary tumors" OR "Nonfunctioning pituitary tumor" OR "nonfunctioning pituitary" OR "non functioning pituitary") OR ((pituitary adenoma OR pituitary adenomas OR pituitary macroadenoma OR pituitary macroadenomas OR pituitary macro-adenoma OR pituitary macro-adenomas OR pituitary tumor OR pituitary tumors OR pituitary tumour OR pituitary tumours OR pituitary neoplasms) AND ("non functioning" OR nonfunctioning))))) AND (vision OR visual system parameters OR ("visual acuity" OR "visual fields" OR "visual field recovery" OR "visual field" OR "visual disturbance" OR "visual disturbances" OR "VF assessment" OR "VF recovery" OR (VF AND eyes) OR "visual acuity" OR "VF deficit") OR visual disorder OR ("Humphrey Field Analyzer" OR "visual outcome" OR "Visual parameters" OR "Visual parameter" OR "visual prognosis" OR vision **OR visual function* OR visual loss***)) AND ((Pre-existing OR postoperatively OR post-operatively OR "time course" OR "predictive factors" OR "predictive factor" OR "early intervention") OR prediction OR predictive value OR "before and after" OR timing OR time **OR ("years after" OR "year after" OR "months after" OR "month after" OR "weeks after" OR "week after" OR "days after" OR "day after" OR "before and after")**))) OR ((("non functioning pituitary adenomas" OR "non functioning pituitary adenoma" OR "Non-functioning pituitary tumours" OR "Non-functioning pituitary tumour" OR "Non-functioning pituitary tumors" OR "Non-functioning pituitary tumor" OR "nonfunctioning pituitary adenomas" OR "nonfunctioning pituitary adenoma" OR "Nonfunctioning pituitary tumours" OR "Nonfunctioning pituitary tumour" OR "Nonfunctioning pituitary tumors" OR "Nonfunctioning pituitary tumor" OR "nonfunctioning pituitary" OR "non functioning pituitary" OR ((pituitary adenoma OR pituitary adenomas OR pituitary macroadenoma OR pituitary macroadenomas OR pituitary macro-adenoma OR pituitary macro-adenomas OR pituitary tumor OR pituitary tumors OR pituitary tumour OR pituitary tumours OR pituitary neoplasms) AND ("non functioning" OR nonfunctioning))) AND (timing OR time **OR ("years after" OR "year after" OR "months after" OR "month after" OR "weeks after" OR "week after" OR "days after" OR "day after" OR "before and after")**) AND (surgery OR surgical* OR surgeon* OR operat* OR surgery))) OR (((("non functioning pituitary adenomas" OR "non functioning pituitary adenoma" OR "Non-functioning pituitary tumours" OR "Non-functioning pituitary tumour" OR "Non-functioning pituitary tumors" OR "Non-functioning pituitary tumor" OR "nonfunctioning pituitary adenomas" OR "nonfunctioning pituitary adenoma" OR "Nonfunctioning pituitary tumours" OR "Nonfunctioning pituitary tumour" OR "Nonfunctioning pituitary tumors" OR "Nonfunctioning pituitary tumor" OR "nonfunctioning pituitary" OR "non functioning pituitary" OR ((pituitary adenoma OR pituitary adenomas OR pituitary macroadenoma OR pituitary macroadenomas OR pituitary macro-adenoma OR pituitary macro-adenomas OR pituitary tumor OR pituitary tumors OR pituitary tumour OR pituitary tumours OR pituitary neoplasms) AND ("non functioning" OR nonfunctioning))) OR hypophysis tumor OR ("Pituitary tumor" OR "Pituitary tumors" OR "Pituitary tumour" OR "Pituitary tumours" OR "Pituitary adenoma" OR "Pituitary adenomas" OR "sellar tumor" OR "sellar tumors" OR "sellar tumour" OR "sellar tumours" OR "sellar neoplasm" OR "sellar neoplasms") OR (sellar.ti AND adenoma.ti) OR (sellar.ti AND adenomas.ti) OR ("suprasellar tumor" OR "suprasellar tumors" OR "suprasellar tumour" OR "suprasellar tumours" OR "suprasellar neoplasm" OR "suprasellar neoplasms") OR (suprasellar.ti AND adenoma.ti) OR ("suprasellar adenomas" OR "pituitary gland adenoma" OR "pituitary gland adenomas" OR "pituitary gland tumor" OR "pituitary gland tumors" OR "pituitary gland tumour" OR "pituitary gland tumours" OR "pituitary gland neoplasm" OR "pituitary gland neoplasms")) AND (timing OR time OR (**"years after" OR "year after" OR "months after" OR "month after" OR "weeks after" OR "week after" OR "days after" OR "day after" OR "before and after")**) AND (surgery OR surgical* OR surgeon* OR surgery OR operat*))) OR (((((transsphenoidal surgery OR "transsphenoidal surgery" OR "transcranial surgery" **OR operat* OR "surgery"**) AND (hypophysis adenoma OR (pituitary adenomas OR pituitary adenoma OR pituitary macroadenomas OR pituitary macroadenoma) OR hypophysis tumor OR ("non functioning pituitary adenomas" OR "non functioning pituitary adenoma" OR "Non-functioning pituitary tumours" OR "Non-functioning pituitary tumour" OR "Non-functioning pituitary tumors" OR "Non-functioning pituitary tumor" OR "nonfunctioning pituitary adenomas" OR "nonfunctioning pituitary adenoma" OR "Nonfunctioning pituitary tumours" OR "Nonfunctioning pituitary tumour" OR "Nonfunctioning pituitary tumors" OR "Nonfunctioning pituitary tumor" OR "nonfunctioning pituitary" OR "non functioning pituitary") OR ((pituitary adenoma OR pituitary adenomas OR pituitary macroadenoma OR pituitary macroadenomas OR pituitary macro-adenoma OR pituitary macro-adenomas OR pituitary tumor OR pituitary tumors OR pituitary tumour OR pituitary tumours OR pituitary neoplasms) AND ("non functioning" OR nonfunctioning))))) AND (vision OR visual system parameters OR ("visual acuity" OR "visual fields" OR "visual field recovery" OR "visual field" OR "visual disturbance" OR "visual disturbances" OR "VF assessment" OR "VF recovery" OR (VF AND eyes) OR "visual acuity" OR "VF deficit") OR visual disorder OR ("Humphrey Field Analyzer" OR "visual outcome" OR "Visual parameters" OR "Visual parameter" OR "visual prognosis" OR vision **OR visual function* OR visual loss***)) AND ("follow-up" OR followup* OR "Follow-Up") AND ("timing" OR "Time" OR "years after" OR "year after" OR "months after" OR "month after" OR "weeks after" OR "week after" OR "days after" OR "day after" OR "before and after" **OR "time"**))))

1 = 0 refs

(("transsphenoidal surgery" OR "transcranial surgery") AND (hypophysis adenoma OR pituitary adenomas OR pituitary adenoma OR pituitary macroadenomas OR pituitary macroadenoma OR hypophysis tumor OR "non functioning pituitary adenomas" OR "non functioning pituitary adenoma" OR "Non-functioning pituitary tumours" OR "Non-functioning pituitary tumour" OR "Non-functioning pituitary tumors" OR "Non-functioning pituitary tumor" OR "nonfunctioning pituitary adenomas" OR "nonfunctioning pituitary adenoma" OR "Nonfunctioning pituitary tumours" OR "Nonfunctioning pituitary tumour" OR "Nonfunctioning pituitary tumors" OR "Nonfunctioning pituitary tumor" OR "nonfunctioning pituitary" OR "non functioning pituitary" OR ((pituitary adenoma OR pituitary adenomas OR pituitary macroadenoma OR pituitary macroadenomas OR pituitary macro-adenoma OR pituitary macro-adenomas OR pituitary tumor OR pituitary tumors OR pituitary tumour OR pituitary tumours OR pituitary neoplasms) AND ("non functioning" OR nonfunctioning))))

((vision OR visual parameter* OR "visual acuity" OR "visual fields" OR "visual field recovery" OR "visual field" OR "visual disturbance" OR "visual disturbances" OR "VF assessment" OR "VF recovery" OR (VF AND eyes) OR "visual acuity" OR "VF deficit" OR "visual disorder*" OR "Humphrey Field Analyzer" OR "visual outcome" OR "Visual parameters" OR "Visual parameter" OR "visual prognosis" OR vision) AND (Pre-existing OR postoperatively OR post-operatively OR "time course" OR "predictive factors" OR "predictive factor" OR "early intervention" OR predict* OR "before and after" OR timing OR time **OR "years after" OR "year after" OR "months after" OR "month after" OR "weeks after" OR "week after" OR "days after" OR "day after" OR "before and after")**)

2. = 2

(("non functioning pituitary adenomas" OR "non functioning pituitary adenoma" OR "Non-functioning pituitary tumours" OR "Non-functioning pituitary tumour" OR "Non-functioning pituitary tumors" OR "Non-functioning pituitary tumor" OR "nonfunctioning pituitary adenomas" OR "nonfunctioning pituitary adenoma" OR "Nonfunctioning pituitary tumours" OR "Nonfunctioning pituitary tumour" OR "Nonfunctioning pituitary tumors" OR "Nonfunctioning pituitary tumor" OR "nonfunctioning pituitary" OR "non functioning pituitary" OR ((pituitary adenoma OR pituitary adenomas OR pituitary macroadenoma OR pituitary macroadenomas OR pituitary macro-adenoma OR pituitary macro-adenomas OR pituitary tumor OR pituitary tumors OR pituitary tumour OR pituitary tumours OR pituitary neoplasms) AND ("non functioning" OR nonfunctioning))) AND (timing OR time **OR "years after" OR "year after" OR "months after" OR "month after" OR "weeks after" OR "week after" OR "days after" OR "day after" OR "before and after"**) AND (surgery OR surgical* OR surgeon*))

3. = 1

(("non functioning pituitary adenomas" OR "non functioning pituitary adenoma" OR "Non-functioning pituitary tumours" OR "Non-functioning pituitary tumour" OR "Non-functioning pituitary tumors" OR "Non-functioning pituitary tumor" OR "nonfunctioning pituitary adenomas" OR "nonfunctioning pituitary adenoma" OR "Nonfunctioning pituitary tumours" OR "Nonfunctioning pituitary tumour" OR "Nonfunctioning pituitary tumors" OR "Nonfunctioning pituitary tumor" OR "nonfunctioning pituitary" OR "non functioning pituitary") AND (timing OR time OR **"years after" OR "year after" OR "months after" OR "month after" OR "weeks after" OR "week after" OR "days after" OR "day after" OR "before and after")** AND (surgery.mp OR surgical* OR surgeon*))

TS=(((pituitary adenoma OR pituitary adenomas OR pituitary macroadenoma OR pituitary macroadenomas OR pituitary macro-adenoma OR pituitary macro-adenomas OR pituitary tumor OR pituitary tumors OR pituitary tumour OR pituitary tumours OR pituitary neoplasms) AND ("non functioning" OR nonfunctioning)) AND (timing OR time OR **"years after" OR "year after" OR "months after" OR "month after" OR "weeks after" OR "week after" OR "days after" OR "day after" OR "before and after")** AND (surgery.mp OR surgical* OR surgeon*))

TS=(("Pituitary tumor" OR "Pituitary tumors" OR "Pituitary tumour" OR "Pituitary tumours" OR "Pituitary adenoma" OR "Pituitary adenomas" OR "sellar tumor" OR "sellar tumors" OR "sellar tumour" OR "sellar tumours" OR "sellar neoplasm" OR "sellar neoplasms" OR "sellar adenoma*" OR "suprasellar tumo*" OR "suprasellar tumour" OR "suprasellar tumours" OR "suprasellar neoplasm" OR "suprasellar neoplasms" OR "suprasellar adenoma*" OR "pituitary gland adenoma" OR "pituitary gland adenomas" OR "pituitary gland tumor" OR "pituitary gland tumors" OR "pituitary gland tumour" OR "pituitary gland tumours" OR "pituitary gland neoplasm" OR "pituitary gland neoplasms") AND (timing OR time OR **"years after" OR "year after" OR "months after" OR "month after" OR "weeks after" OR "week after" OR "days after" OR "day after" OR "before and after")** AND (surgery.mp OR surgical* OR surgeon*))

**CINAHL**

<http://search.ebscohost.com/login.aspx?authtype=ip,uid&profile=ehost&defaultdb=cin20>

**PsycINFO**

<http://search.ebscohost.com/login.aspx?authtype=ip,uid&profile=ehost&defaultdb=psyh>

**Academic Search Premier [fulltextzoeken]**

<http://www.ebscohost.com/thisTopic.php?marketID=1&topicID=1>

Journal databases [fulltextzoeken]

**A. ScienceDirect**

<http://www.sciencedirect.com/science?_ob=MiamiSearchURL&_method=requestForm&_temp=all_boolSearch.tmpl&_acct=C000026638&_version=1&_urlVersion=1&_userid=530453&md5=d44bd9fa9076bb9b258a588b309be1e3>

**B. Springer**

<http://springerlink.metapress.com/app/home/search-citations.asp?wasp=3pvxje0mwm6qxm8d2hw3>

**C. Wiley-Blackwell**

<http://www3.interscience.wiley.com/cgi-bin/simplesearch>

**D. LWW**

<http://ovidsp.ovid.com/ovidweb.cgi?T=JS&PAGE=main&MODE=ovidclassic&D=ovft>

**E. Highwire**

<http://highwire.stanford.edu/>

**F. Taylor & Francis/Informaworld**

<http://informahealthcare.com/>

**G. Google Scholar**

<http://scholar.google.com/>

**________________________________________________________________________**

Terminology help:

- <http://conceptwiki.org/index.php/Main%20Page>
- <http://www.lumc.nl/rep/cod/redirect/1060/ovb/ov_terminologie.html>
